# Exploring the Frontiers of Food Science: A Comprehensive Review of Advanced Magnetic Resonance Applications in Food Analysis, Quality Analysis, and Safety Assessment

**DOI:** 10.1002/fsn3.70643

**Published:** 2025-07-20

**Authors:** Zina T. Alkanan, Ammar B. Altemimi, Farhang H. Awlqadr, Qausar Hamed Alkaisy, Seyed Mohammad Bagher Hashemi, Anubhav Pratap‐Singh

**Affiliations:** ^1^ Department of Food Science, College of Agriculture University of Basrah Basrah Iraq; ^2^ Department of Food Science and Technology, Faculty of Agriculture University of Tabriz Tabriz Iran; ^3^ Department of Food Science and Quality Control Halabja Technical College, Sulaimani Polytechnic University Sulaymaniyah Iraq; ^4^ Department of Dairy Science and Technology, College of Food Sciences University of AL‐Qasim Green Al Qasim Iraq; ^5^ Department of Food Science and Technology, Faculty of Agriculture Fasa University Fasa Iran; ^6^ Dan on Food and Beverage Innovation Center The University of British Columbia Vancouver British Columbia Canada

**Keywords:** artificial intelligence (AI) in food analysis, food authentication, foodomics, magnetic resonance, nuclear magnetic resonance

## Abstract

Magnetic resonance (MR) technologies, such as nuclear magnetic resonance (NMR), magnetic resonance imaging (MRI), and electron spin resonance (ESR), have been identified as fundamental tools in the modern food science, which allows virtually high precision and non‐invasive detection during quality assessment, safety analysis, and authenticity verification. This path reveals the adaptability of MR technologies and their importance across various food aspects. The article emphasizes their utilization in ensuring proper composition, detecting fraudulent elements, and monitoring changes during processing and storage in different food products. The application of MR techniques in the analysis of the dairy and cheese industries, has indeed proven to be a necessity in the profiling of metabolites, analyzing ripening stages, and antioxidant stability assessment, when coupled with quality control and regulatory compliance. Moreover, MR AI driven food analysis propels the development of AI, which supports functionality with high processing speeds, prediction modeling, and quick safety diagnostics. These new technologies do not only simplify food authentication and traceability but they also contribute to sustainability and customer transparency. The review, by displaying the key breakthroughs and the possibilities, keeps pace with the main role of MR technologies in food science, which is the driving force behind innovation and the meeting of global food systems ever‐changing needs.

## Graphical abstract

The paper reviews advanced magnetic resonance (MR) technologies—NMR, MRI, ESR, and MRS—for food analysis, quality, and safety. MR techniques enable non‐invasive detection of adulteration, compositional profiling, and monitoring of food processing and storage. Integrating MR with AI enhances real‐time diagnostics, predictive modeling, and food authenticity, supporting innovation and sustainability.

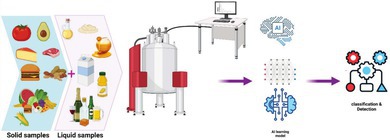

## 1 Introduction

The application of high‐resolution nuclear magnetic resonance (NMR) spectroscopy, along with magnetic resonance imaging (MRI) and electron paramagnetic resonance (EPR), has been a major breakthrough in food science research and product development. These methods, some of them being non‐intrusive, very sensitive, and versatile in the spectrum of applications, are now unfathomably vital tools for the analysis of food chemistry, the determination of quality, and the provision of overall safety (Qu and Jin 2022). Magnetic resonance methods are very useful in identifying the chemical composition and structural stability of food. Their unique ability to afford micro and macro‐structural and molecular insights is unmatched. In the simplest terms, NMR‐based metabolomics is a pioneering method for food evaluation, which allows researchers to analyze food products from the farm to the dinner table, as well as during digestion (Chaudhary et al. 2023). Such inquiries give us not only a better knowledge of nutritional properties but also are responsible for sustainable practices by recycling bio‐waste into functional food components (Ciampa et al. 2022). The use of MR techniques, NMR, and EPR has stood out due to their remarkable performance in plant‐derived natural constituents. The procedures involved not only empower the precise revelation of bioactive compounds like antioxidants, antimicrobials, and other phytochemicals but also include nuclear magnetic resonance; further, they ensure a wider dynamic range and offer minimal sample preparation. It accomplishes this in a more preponderant way than, for example, the traditional chromatography and mass spectrometry that require time and a lot of work to remove the sample. This new development proves the importance of the technique in the safety and quality of the food supply, but also opens the door to the discovery of new drugs and the development of nutraceuticals (Anand et al. 2022). NMR spectroscopy, a system that also influences the quality indicators of food oils, namely, palm and olive oil, has been empowered as well. This technique, used in combination with chemometric analysis, effectively sped up the process profiling of the chemical parameters, which in turn improved traceability and authenticity of the verification processes (Shi et al. 2023). These developments are equipped with strong instruments for detecting food adulteration and ensuring that all food safety standards are met (Maestrello et al. 2022). Magnetic resonance techniques *are* cutting‐edge tools in detecting foodborne diseases and pollutants with great accuracy. The non‐destructive technologies, through hyperspectral and multispectral imaging, as well as the chemometric techniques, are based on MR, which are increasingly being used for food analysis to find fast and cost‐effective ways. These MR‐based methods not only ensure a high level of food safety, but they also alleviate environmental issues associated with older chemical testing techniques—for example, reducing the hazardous waste and solvent use generated by traditional assays (Kharbach et al. 2023). The recent study mentioned that the MR technique can analyze the intricate food ingredients and compounds that are ubiquitous in food. For such purposes, the scientists resorted to magnetic resonance (NMR) spectroscopy to delve into the fiber structure of their diet, the symbiosis of gut flora, and the metabolic effects of different food regimens. It is more than evident that having an understanding of these topics is priceless due to the successful development of food products that are not only healthier but also customized to the needs of the people (Bertram 2022). Developments in magnetic resonance have also brought forth autonomous and complete time‐continuous assessment of food safety characteristics. Methods such as singlet state‐filtered NMR make it possible to accurately follow the metabolic changes occurring within the animal organisms facing foodborne toxins. Such practices serve as a guide to the ability we have as humans to understand and prevent the toxicological impact on food buyers (Osheter et al. 2022). For instance, advanced NMR/MRI methods can measure salt content (e.g., sodium‐23 (^23^Na) MRI tracking sodium ions), along with pH and water activity, which has opened up a new field of predictive microbiology by monitoring these factors in foods (Li et al. 2023). Nevertheless, Artificial Intelligence, also known as AI, combined with outstanding analytical technology, is rapidly changing various scientific domains, such as food science (Esmaeily et al. 2023). AI algorithms, especially in machine learning and deep learning, can analyze complex magnetic resonance data to extract the required information from NMR and MRI signals (Dong 2024). The food authenticity, safety monitoring, and adulteration detection accuracy and efficiency are being enriched by AI‐augmented high‐throughput analysis, predictive modeling, and real‐time quality evaluation. AI‐driven pattern recognition can uncover compounds in food that traditional spectral analysis struggles to detect. By learning the subtle patterns in NMR/MR spectra, AI helps assign resonance peaks to specific substances and distinguishes among complex sample profiles (e.g., differentiating authentic products from adulterated ones; Gbashi and Njobeh 2024). In conclusion, the infusion of magnetic resonance‐based tools in food science is recovering the shape of quality control, safety assessment, and nutritional research. These accomplishments not only respond to the customer's imperative to have food honesty and safety but also let the boundaries of technology be broken in the field. Deferral, effectuation, and absence of bodily injury rank MR methods as the necessary instruments in the yet to come, as far as the environment is concerned. This paper aims to illustrate the cross‐cutting propositions of high‐tech magnetic resonance (MR) technologies in food science, with special reference to their functions in food chemistry, quality analysis, and safety evaluation. The paper describes the contribution of MR technologies to the solution of key problems such as counterfeiting, the detection of contaminants, and sustainability, as well as giving an overview of the potential for the subject to bring about new scientific discoveries and future applications.

## 2 Types of Magnetic Resonance (MR) Used in Food

**FIGURE 1:**
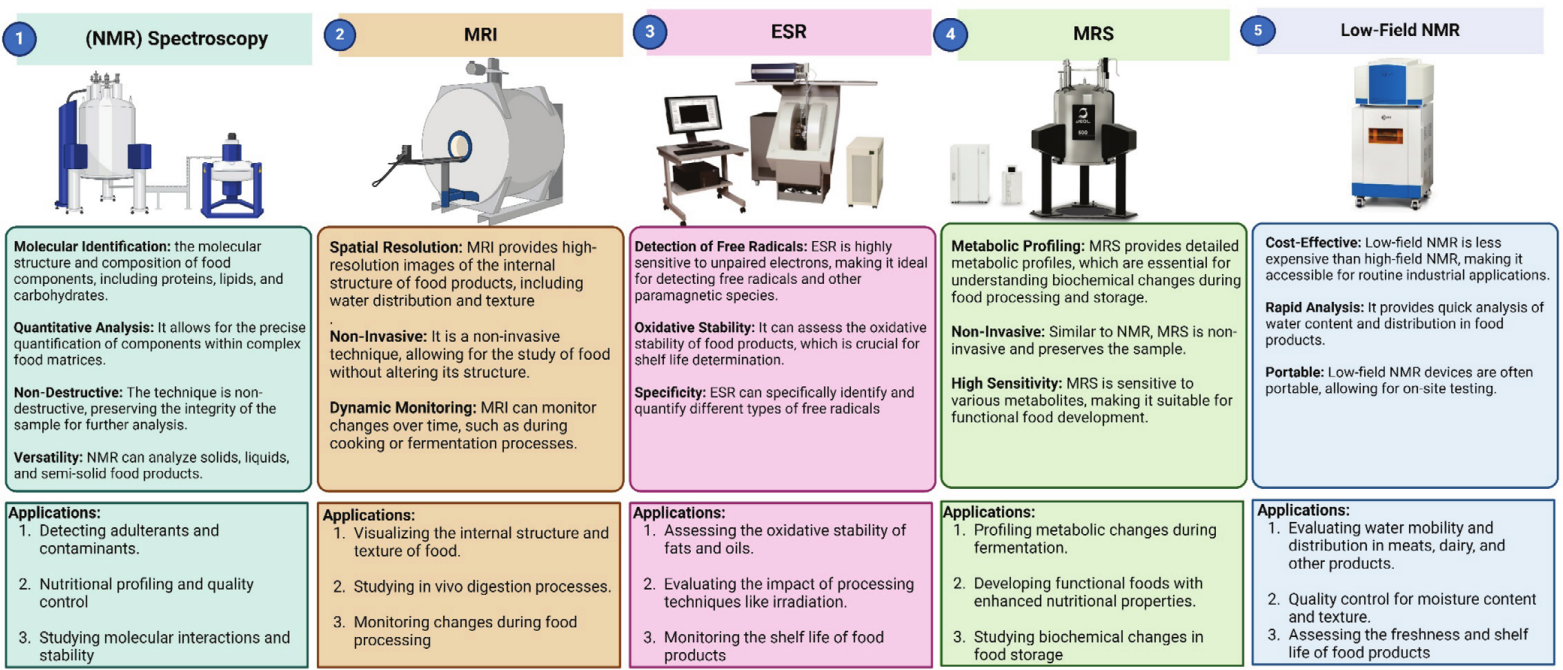
Characterization of magnetic resonance (MR) techniques used in food applications.

Magnetic Resonance (MR) is the technology used in MR devices such as NMR, MRI, ESR, MRS, and Low‐Field NMR. High‐field and low‐field NMR, MRI and, ESR are among the different MR techniques that are available, each of which has a different purpose in food processing. It should be noted that the term “Magnetic Resonance Spectroscopy (MRS)” is often used interchangeably with NMR spectroscopy; both share the same physical basis of nuclear magnetic resonance, differing mainly in context (e.g., in vivo or MRI‐based spectroscopic analyses vs. conventional laboratory NMR), food analysis, quality evaluation, and safety control are the primary tasks of these technologies. In Figure 1, the technologies are contrasted and, it is evident that they have distinct functions such as food analysis, quality assessment, and safety monitoring. TD‐NMR is the name for low‐field NMR, which is a technique that includes both time‐domain (TD‐NMR) and frequency‐domain NMR instruments, but they are coming to the field with different applications. Time‐domain NMR (TD‐NMR) makes it possible to quickly and non‐destructively assess food quality. For example, TD‐NMR can detect a food's moisture content and distribution, fat content, and texture properties, providing a fast evaluation of various products (Colnago et al. 2021). On the other hand, the frequency‐domain benchtop NMR systems have the same operating principle as high‐field spectrometers, but in an external magnetic field that is lower, they can perform molecular characterization at a lower cost and thus be less complex (Yu et al. 2021). These benchtop NMR systems have become valuable for on‐site food authentication and routine structural analysis of foods, and are especially useful for detecting adulterants and monitoring freshness in products (McVey et al. 2021). Magnetic Resonance Spectroscopy (MRS), the main application of high‐field NMR, is known to have the best spectral resolution and chemical specificity, and we can say that this is the essential instrument for metabolomics and detailed molecular profiling in food science. Hence, to accurately define the analytical capabilities of MR techniques in food research, a distinction of low‐ and high‐field (respectively, TD‐NMR and benchtop NMR) and high‐field NMR is indispensable (Hara et al. 2022). High‐field NMR, while offering excellent spectral resolution, often requires extensive sample preparation (such as diluting in deuterated solvents or extracting components). This level of sample manipulation means that, although NMR is largely non‐destructive, high‐field implementations are more invasive than low‐field NMR or MRI in practice (Castaing‐Cordier et al. 2021). A key distinction of high field NMR, being the capability to do direct, in situ investigation that can be done without major sample preparation like in TD‐NMR or MRI, is the most critical when comparing high‐field NMR with time‐domain NMR (TD‐NMR) and MRI, which allow for the direct, in situ analysis without a significant sample preparation, respectively. This is why, while high‐field NMR presents excellent spectral resolution for food authentication, adulteration detection, and metabolomics, we can still describe it as a less invasive, but not purely non‐destructive techniqu (Qu and Jin 2022). In recent years, food scientists have rapidly adopted NMR spectroscopy as a tool for food analysis because it is non‐destructive and offers high analytical resolution. The continuous technological developments in NMR greatly increase our ability to check the quality and safety of food. Such progress is a significant achievement in the field of food chemistry that introduces a good number of new NMR‐based methodologies for the assessment of food quality. One emerging approach involves analyzing the complex chemical makeup of food components like how one would deconstruct the bouquet of a perfume. Developing methods to profile all these flavor and aroma constituents has been a crucial innovation, leading to many scientific and practical applications (Fan and Zhang 2019). This technology has also used diet assessment, which recognizes metabolite alterations related to certain dietary habits, thus increasing our knowledge of food's effects on gut function and metabolism (Bertram 2022). Magnetic resonance imaging (MRI) is another advanced MR technology used to look into the structure of food with great resolution non‐invasively, without tampering with it. Thus, it is appropriate for studying water distribution, structure, and the texture changes during processing. MRI, a non‐invasive technique, is utilized in documenting food as it cooks or ferments, and similarly in research on digestion. By being able to dynamically monitor, MRI engineers can observe the process development in real time and gain important results of what processing influences the food structure and quality (Ripoli et al. 2021). Because MRI can non‐invasively monitor key food quality factors, enhancing food safety measures directly. In fact, MRI's ability to reveal internal changes in food gives it predictive power in food safety research—helping anticipate spoilage or contamination before they become critical. The use of ESR is crucial in food and nutrition science for determining the oxidative stability of fats and oils that are important for food storage. ESR's ability to detect free radicals enables it to become a category term for the very oxidizing stress markers, and it is, for example, a very detailed analysis of the influence of food stabilization using irradiation. The precision of these oxidative markers through ESR takes the food to another level in terms of quality assessment by demonstrating the ability to identify the early degradation processes, ultimately improving both the control and shelf life of the food (Barba et al. 2020). Magnetic resonance spectroscopy (MRS) is a method that uses NMR's principles to analyze metabolism, but it is optimized for metabolic profiling. Increased sensitivity of MRS to various metabolites enables the monitoring and analysis of the biochemical changes in foods during their processing and preservation. The precision of this instrument has made MRS one of the most advanced instruments in metabolic profiling. Thanks to MRS, it was possible to visualize the metabolic shifts during the fermentation process and explore those of the functional components responsible for food nutrition. By identifying the effect of antioxidants and prebiotics on the quality of consumers' food and their nutrition, MRS is advantageous for developing functional foods (Ciampa et al. 2022). Low‐field NMR is an inexpensive and portable alternative to high‐field NMR, thus providing rapid quality measurements in industrial places, which are also quite affordable. It does a rapid analysis of moisture content, which helps in tracking the moisture, texture, and other factors that are important for the product which ultimately does the real‐time quality control for things like dairy and meat. This specific feature is easily transportable and irreplaceable in the industrial food production sector, where it facilitates on‐site testing and, consequently, immediate changes to ensure product quality. In the latest developments, the technology of low‐field NMR has shown it can be used for tracking the moisture level and the texture of food, which makes it more reliable and useful for food quality and food safety (Yu et al. 2021).

In recent advancements, Artificial intelligence (AI) is broadly utilized in magnetic resonance (MR) technologies, especially NMR, for dealing with analysis issues and analyzing big, intricate datasets. The AI‐supported NMR methods are based on machine learning algorithms for easy and quick interpretation of the spectra such that the detection of food components, contaminants, and subtle quality variations becomes more accurate and faster. Such adaptation also extends the NMR capabilities in food identity verification, adulteration detection, and freshness determination that are sensitive and efficient types of investigations (Kuhn 2022; Kuhn et al. 2024). In the context of foodomics, AI‐powered NMR analysis supports the development of predictive models that correlate molecular profiles with nutritional value and health implications. These models are crucial for evaluating the role of diet in health and optimizing the design of functional foods (Kuhn 2022). Ultimately, the collaboration of AI and NMR undoubtedly paves the way for a range of productive, scalable, and smart food analysis systems, which greatly strengthen innovative capabilities in the areas of safety, quality, and nutrition.

## 3 Fundamentals of MR Instruments for Food Applications

Usually, magnetic resonance (MR) instruments are chiefly utilized in food science because of their capability of executing non‐invasive and minimally invasive analyses of foods. Each MR system is designed to have particular parts that are made according to the needs of the analytical methods for food applications. Nonetheless, their achievement depends on the resolution, sensitivity, sample preparation requirements, and cost‐effectiveness.

### 3.1 NMR Spectroscopy

**FIGURE 2:**
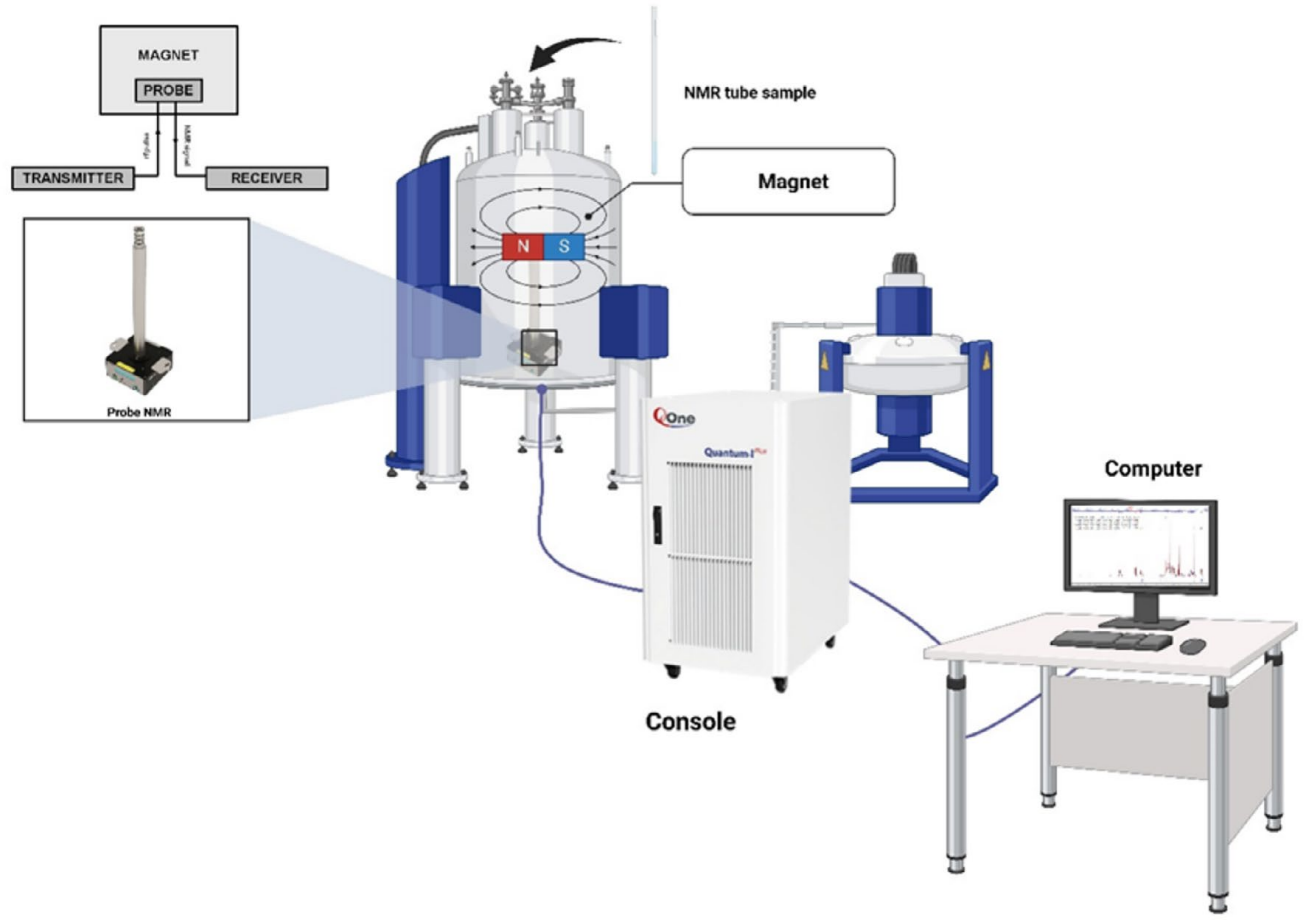
NMR spectroscopy instruments.

The major part of (NMR) nuclear magnetic resonance spectroscopy equipment is made up of a superconducting magnet, which produces a really powerful magnetic field essential for aligning the nuclear spins of sample molecules. A probe, containing coils for transmitting and receiving radiofrequency (RF) signals, holds the sample shown in Figure 2. The RF transmitter and receiver send and detect radiofrequency (RF) signals interacting with the sample's nuclear spins, inducing resonance at characteristic frequencies. The RF transmitter generates precisely tuned radiofrequency pulses that excite the nuclear spins of the sample placed inside the magnet. Once the excitation occurs, the RF receiver detects the emitted signals as the nuclei return to equilibrium. These signals are weak and require amplification before being processed into readable NMR spectra. The accuracy of RF pulse control directly influences spectral resolution and sensitivity, which is crucial for detecting molecular components in food (Dong et al. 2023). Pulse programmers coordinate RF pulses and delays, which dictate the NMR experiment type. It modifies pulse sequences like spin‐echo sequences in relaxation studies for food texture research or decoupling techniques to remove spin–spin interactions in high‐resolution food compositional studies. Advanced multi‐dimensional NMR investigations, such as 2D‐NMR for food chemical fingerprinting, require accurate pulse programming timing (Lhoste et al. 2022). For computational analysis, NMR signals must be transformed from analog to digital. Even weak signals from low‐concentration food components can be accurately converted by the console's analog‐to‐digital converter (ADC). The ADC sample rate and resolution affect spectrum clarity, especially for food trace chemicals and pollutants (Koczor and Rohonczy 2015). After digitization, the console processes the raw data using the Fourier transform (FT) algorithms, converting time‐domain signals into frequency‐domain spectra that scientists can interpret. This step is crucial for identifying chemical compounds based on their characteristic resonance frequencies. Additionally, advanced data analysis techniques such as principal component analysis (PCA) and chemometric modeling can be integrated into the console software to enhance food authentication, adulteration detection, and quality assessment (Beć et al. 2022). A computer system controls the instrument and analyzes the data, making NMR spectroscopy a multifaceted technology for molecular determination, quantification, and analysis of various food components (Bai 2008). NMR is extensively used for food analysis, particularly in profiling lipids, carbohydrates, and metabolites. However, the identification and quantification of proteins using NMR are typically conducted on purified protein samples for structural characterization studies, rather than in complex food matrices (Qu and Jin 2022). It allows for the detailed study of food composition, including the detection of adulterants and contaminants (Consonni and Cagliani 2022). Unlike chromatographic methods, NMR is inherently reproducible and does not require complex calibration standards, making it ideal for routine food quality control (Parlak and Güzeler 2016). Despite its strengths, NMR requires significant sample preparation, particularly for high‐field NMR, which demands deuterated solvents for solution‐state analysis. This requirement raises concerns regarding its classification as a non‐destructive technique (Beteinakis et al. 2022). Also, NMR instruments are costly and need experts to handle them, so this is the reason small‐scale food producers or developing countries can rarely access them. Besides, the instrument sensitivity for the actual contamination is relatively low but can be increased by a combination of it with hyperpolarization or by the use of cryoprobes (Moraes et al. 2025).

### 3.2 Magnetic Resonance Imaging (MRI)

**FIGURE 3:**
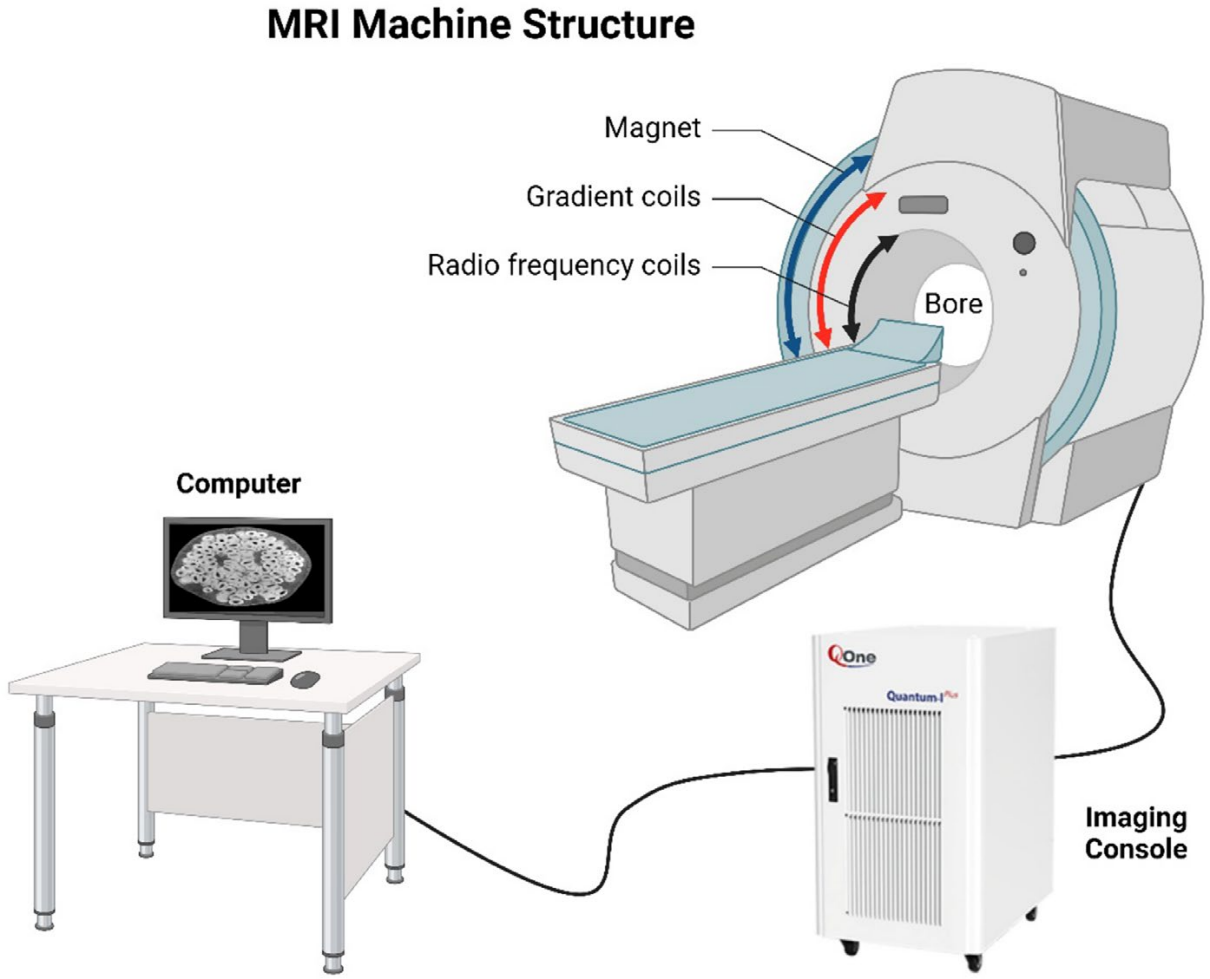
MRI instruments.

The instruments of magnetic resonance imaging (MRI) include a powerful superconducting magnet that creates a 3D magnetic field, gradient coils to spatially encode the MRI signals, and RF coils to transmit and receive RF pulses. The imaging console and computer system work together to process the signals and generate high‐resolution images of the food's internal structure. MRI is a non‐abrasive imaging technique that helps to visualize the internal structure and texture of the food without any alterations. It is an incredibly beneficial tool for tracking the moisture spread and the integrity of food products such as meat, dairy, fruits, and baked goods (Mohammadi‐Moghaddam et al. 2024). MRI has been helpful for studying the structure and distribution of the gas bubbles in bread dough and it will eventually provide the necessary data to enhance the quality control system of the baking technology (Sun et al. 2023). Moreover, MRI assists during food digestion projects, allowing scientists to trace the different food breakdown processes in the gastrointestinal tract, which is crucial for making functional foods (Musse et al. 2023). An important constraint related to MRI is that it is not as susceptible as high‐field NMR to chemical composition. The most common MRI application is a high‐resolution scan, but it is a tool that cannot be used to measure molecular transformations in food matrices (Moraes et al. 2025). Additionally, MRI is done in large sizes and costs much money, plus it needs to take the large instrumentation, the size of a truck. This makes it impractical for small food analysis (Webb and Obungoloch 2023). Moreover, image processing technology can be very challenging and necessitate cutting‐edge computation tools to derive quantitative information (Dada and Awojoyogbe 2021). The most common MRI sequences produce proton density or relaxation‐weighted images, but advanced MRI can map relaxation parameters T1 and T2 across a sample. These relaxation time maps are quantitative and relate to water mobility and interactions in tissues (Gaeta et al. 2024; Rodriguez et al. 2023). Thereby indicating qualities like moisture content or firmness in foods. For example, regions of a fruit or fish fillet with shorter T2 might correspond to more solid‐like or dehydrated tissue (important for freshness assessment), whereas longer T2 regions indicate juicier or water‐rich zones. MRI's spatial resolution can vary. Standard clinical‐style MRI scanners (horizontal bore, ~1.5–7 T) typically achieve on the order of ~100 μm resolution (Barigou and Douaire 2013). For food samples, which is sufficient for many structural analyses. However, using high‐field vertical‐bore NMR magnets with microimaging probes (sometimes termed magnetic resonance microscopy), resolutions can reach tens of microns (5–40 μm; Barigou and Douaire 2013). Enabling microscopic inspection of food microstructure. Such high‐resolution MRI (μMRI) has been applied to nuts, seeds, and muscle foods to observe microstructural details like oil distribution or muscle fiber organization. The trade‐off is that higher fields and smaller sample coils yield smaller fields of view, so researchers choose the system (horizontal vs. vertical magnet) appropriate to the sample size and resolution needed. MRI can also be combined with spectroscopic techniques. Chemical shift imaging (CSI), or magnetic resonance spectroscopic imaging (MRSI), acquires a full NMR spectrum at each image voxel, thus mapping the spatial distribution of specific chemical compounds. For instance, CSI can differentiate fat and water signals based on their distinct resonance frequencies, allowing the creation of separate “fat fraction” images in foods (Dyke et al. 2018). This approach has been used to quantitatively map fat content in meats and dairy products, exploiting the ~3.5 ppm chemical shift difference between lipid and water protons. Such MR chemical shift imaging provides a powerful way to assess heterogeneity in composition (e.g., marbling in meat or fat distribution in cheese) noninvasively (Dyke et al. 2018). Despite its strengths in structural visualization, conventional MRI has limitations. It generally cannot directly detect trace‐level chemical compounds or minor metabolites due to lower spectral resolution and sensitivity compared to dedicated high‐field NMR spectroscopy. Nonetheless, ongoing advances—such as stronger magnets, improved gradient hardware, and faster imaging sequences—continuously enhance MRI's capability and throughput for food applications. High‐field small‐bore MRI systems and emerging portable MRI technologies are expanding the practicality of MRI in food research by increasing resolution and reducing system footprint (Figure 3).

### 3.3 ESR

**FIGURE 4:**
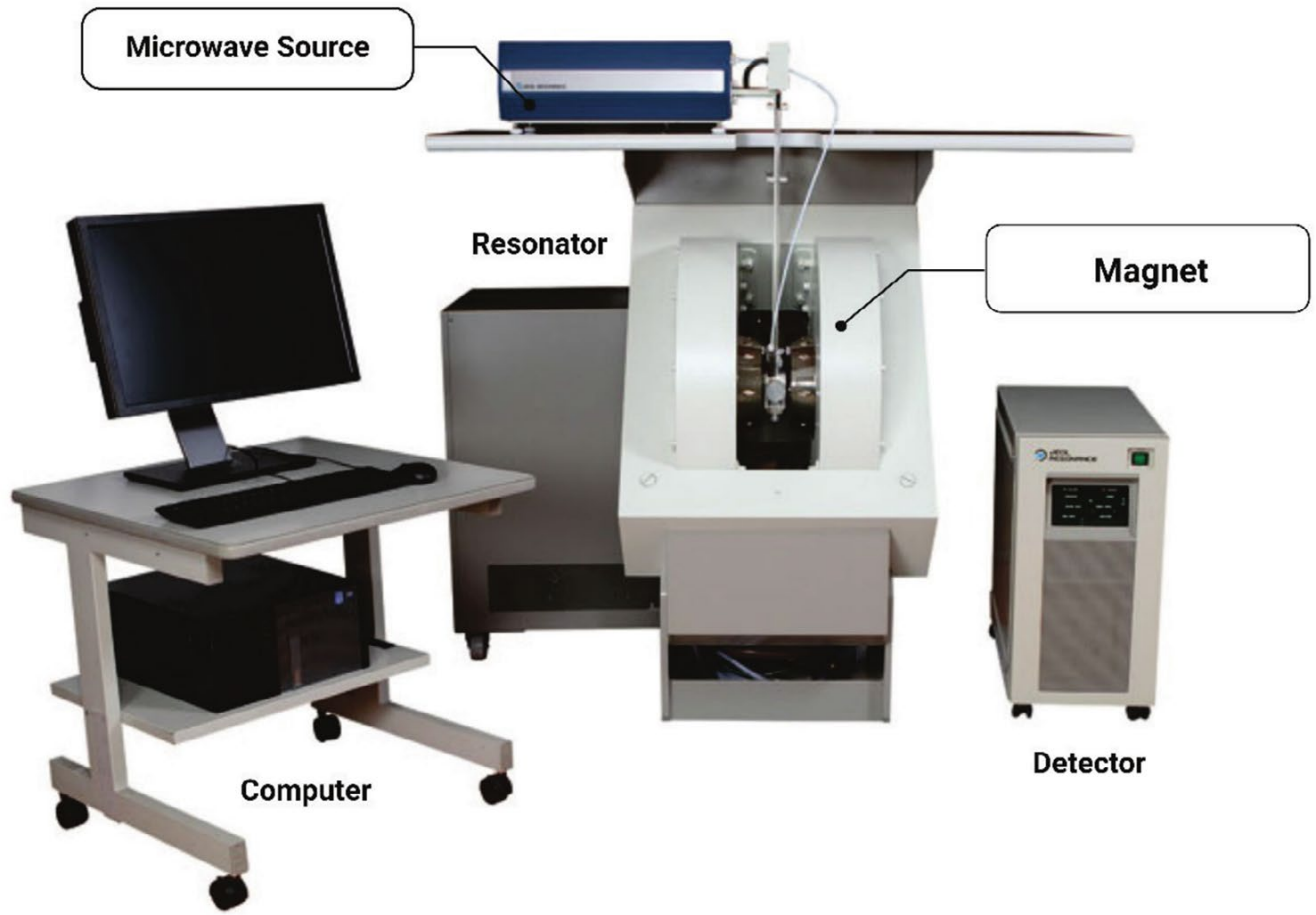
ESR instruments.

Electron spin resonance (ESR) instruments are explicitly conceptualized to look for free radicals in food. These gadgets come with a magnet used to align the unpaired electrons' spins, a microwave source that excites the electrons, a resonator to fit the sample taught in a computer, and a detector to measure microwave absorption. The computer processes the data to produce the spectra, as shown in Figure 4; ESR is used to understand the presence and quantity of the free radicals associated with the oxidation processes (Khan and Shahid 2022). ESR is incredibly adept at disrupting free radicals and thwarting oxidative changes, thus holding tremendous importance as a smoking gun for food freshness evaluation and extension of shelf life. This technology is largely deployed for tracking lipid oxidation occurring in oils and fats, which is not only an important step in quality control in edible oils but also the sole determinant of their shelf life (Kameya 2022). ESR can additionally be used to check the impact of dealing with options, like irradiation, on food quality (Barba et al. 2020). On the other hand, the key drawback of ESR in the field of application is its narrow scope, since it could only be used to detect paramagnetic species and is less capable of analyzing the whole composition of foods (Nilghaz et al. 2022). Besides, the ESR depends on the material's water content; for this reason, the carrot should always be dried or pre‐treated before analysis (Khan and Shahid 2022).

### 3.4 MRS

The devices for magnetic resonance spectroscopy (MRS), which are simply NMR machines, have a magnet for the alignment of the nuclear spins, an RF transmitter and a receiver for sending and detecting pulses, and a computer system for processing MR signals. On the one hand, the principal advantage of MRS over NMR lies in its being used primarily for metabolic studies. The mode of the computer analyzing this process is as follows: it incites metabolic profiles, which in turn give information about biochemical changes, good or bad, in the food from processing to storage (Smeets et al. 2020). MRS can depict explicitly the biochemical changes in food, which can promote the creation of functional foods with enhanced nutritional properties (Hatzakis 2018). MRS in the other lane necessitates high‐field NMR systems, representing cost and complexity problems (Manso Jimeno et al. 2023). The methodology also requires post‐processing and a standardized analysis to interpret metabolic data effectively (Mugler and Jenkins 2023).

### 3.5 NMR Instruments With Low‐Field Capabilities TD‐NMR and Benchtop NMR

**FIGURE 5:**
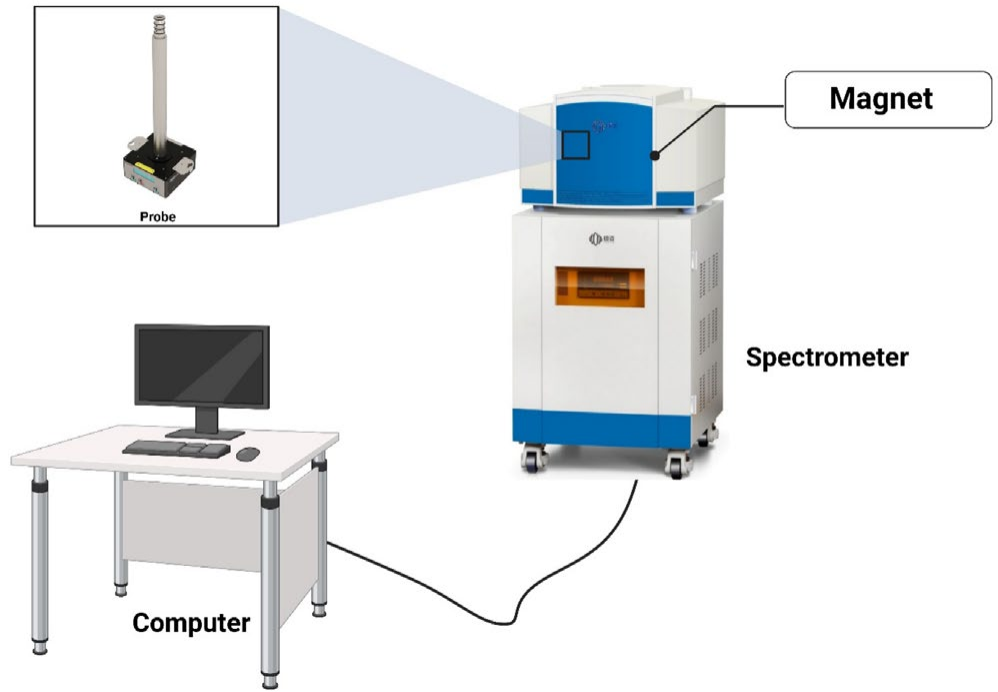
NMR instruments with low‐field capabilities.

Low‐field NMR instruments offer portability and cost‐effectiveness compared to high‐field systems, feature a magnet that generates a lower‐strength magnetic field, a probe with coils for RF transmission and reception, and a computer system for signal processing, as shown in Figure 5. The computer system controls the analysis and interprets the spectra, which indicate water content and distribution. These instruments are widely used for evaluating moisture content and texture in food products, providing quick and efficient quality control (Capitani et al. 2017). In addition, low‐field and conventional NMR have different applications and capabilities, making them appropriate for different food analysis functions. Low‐field NMR uses portable, affordable equipment to measure magnetic fields between 40 and 100 MHz. These tools are ideal for rapid, non‐invasive food quality tests, such as water content, fat distribution, and oil droplet sizes. Their compact size and on‐site assessments make them perfect for industrial quality control. Low‐field NMR is unsuitable for complicated molecular‐level studies due to its poorer sensitivity and resolution (Yao et al. 2022). However, conventional NMR employs high‐field superconducting magnets at 300–1200 MHz. Due to their high resolution and sensitivity, these technologies are essential for complicated food research, including structural elucidation of bioactive chemicals and metabolites in food matrices. Conventional NMR can authenticate food products and research complex food systems, but it is more expensive and requires specialist equipment and knowledge (Teipel 2023; Wikus et al. 2022). Specific needs determine whether food analysis uses low‐field or conventional NMR. Low‐field NMR is useful for quality control, non‐destructive testing, and quick food, water, or oil content monitoring. For high‐resolution studies like identifying unknown substances or examining complicated food system interactions, conventional NMR is preferred. Portable devices and improved sensors have made low‐field NMR technology suitable for more industrial applications. Then, low‐field NMR has lower resolution and sensitivity, making it unsuitable for detailed molecular analysis (Bao et al. 2023). Additionally, it cannot accurately detect trace contaminants or complex metabolites, which limits its applications compared to high‐field NMR (Adels et al. 2024).

## 4 Preparation Foods Samples for Magnetic Resonance

**FIGURE 6:**
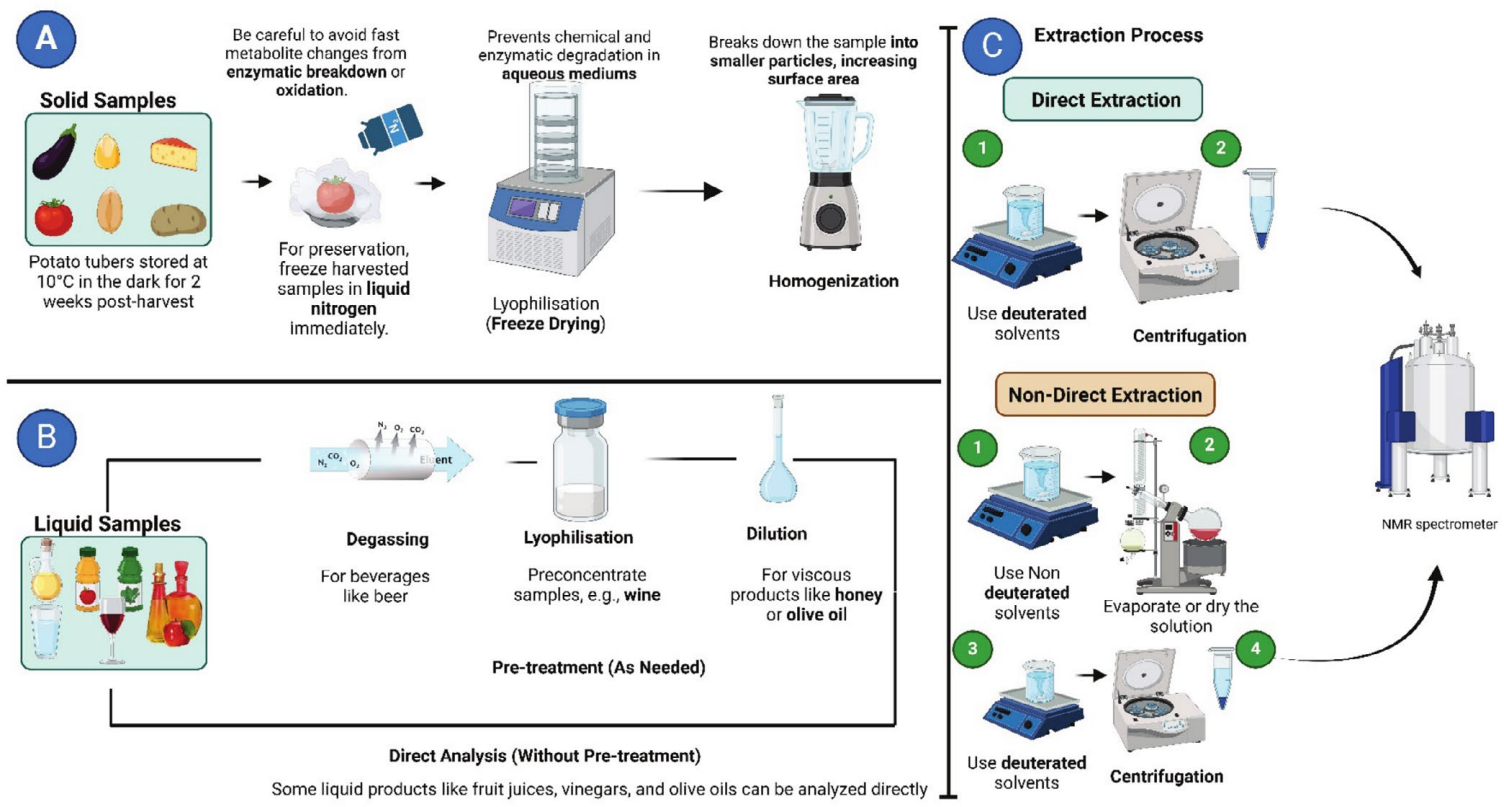
Liquid and solid food samples preparation for NMR analysis: (A) Stages in the preparation of solid samples, involving such steps as freezing, lyophilizing, and homogenizing (B) Liquid sample preparation with possibilities of direct analysis or pre‐treatment steps such as degassing, lyophilization, or dilution (C) The extraction of both direct and non‐direct methods may involve the steps of solvent selection, centrifugation, and drying before NMR analysis.

The process of preparing a food sample is a crucial part of getting the correct and reliable results in the magnetic resonance (MR) analysis, which in turn stands for both nuclear magnetic resonance (NMR) and magnetic resonance imaging (MRI). In this process, a sample has to be optimized to the conditions that provoke resolution and are minimally interfered with, and reproducibility is assured. For liquid‐state NMR, food samples can be homogenized and diluted with deuterium oxide to lock the magnetic field and provide a clear baseline. Additionally, buffer solutions can be opted for pH maintenance, leading to the substance's intactness at the molecular level (Eltemur et al. 2023). Solid food, such as grains, fruits, or meat, may likely be made unusable by water content that can disturb the data analysis, and thus, they often require lyophilization or drying to reduce water content, and thus, the clarity of spectra is undisturbed. Next, samples are turned into powders to make a homogeneous mixture, which results in the enhancement of the sample's uniformity, and the signal's acquisition shows less variability. For high‐definition magic angle spinning (HR‐MAS) NMR, intact tissues or small solid fragments are prepared with as little manipulation as possible so that structurally and compositionally intact samples may deteriorate (Bahri et al. 2023). Magnetic resonance imaging (MRI) is a technique that currently values unadulterated food samples to keep the natural spatial arrangement of elements such as water, lipids, and proteins. Specifically designed holders or containers compatible with MR systems are employed to position the sample without causing disruption. By real‐time tracking, including without extensive manipulation of samples, researchers can visualize molecular structures and interactions and distinguish the structural changes brought about by interactions (Kerr et al. 2022). The choice of solvents and reagents is made considering the analytical object of our interest. For example, the most common ways to extract lipids are using a methanol–water or chloroform–methanol mixture, while the metabolite extraction in aqueous matrices is performed using per chloric acid. Those steps are completed with strict control to avoid the target compounds' deterioration or contamination. This type of procedure is common for researching the fatty, oily, and other food components that are fat‐soluble (Saini et al. 2021). Low‐field NMR and MRI necessitate only a small amount of sample preparation, which makes them perfect for industrial applications demanding a quick study of a product. However, for detailed compositional studies or metabolomic profiling, high‐field NMR demands meticulous preparation, including removing particulates and magnetic impurities that could distort the results. Centrifugation and filtration are often employed during sample preparation to achieve this level of purity (Mal et al. 2021). Multivariate statistical techniques combined with advanced sample preparation protocols enhance the quality of data obtained from MR analyses. When you prepare in detail the methods according to the part of the study, such as structure elucidation, quality assessment, or adulteration detection, the experts in this area are guaranteed that MR can read the complexities of food matrices accurately (Jeppesen and Powers 2023). Figure 6 summarizes the major food products analyzed using MR methods and their respective applications.

### 4.1 High‐Field Strength and Sensitivity Enhancements in NMR Instrumentation

Despite these successes, classical high‐field NMR faces sensitivity limits for trace analysis. NMR detection typically requires microgram‐to‐millimolar concentrations, whereas contaminants may be in the nanogram range. To bridge this gap, several strategies are employed. First, sample enrichment is critical: because contamination levels are low, food samples often undergo extraction or concentration steps (e.g., solid‐phase extraction and lyophilization) to boost the contaminant's concentration into NMR‐detectable ranges. For example, to detect pesticide residues in milk (a complex, high‐fat matrix), one might extract the residues into a small volume of deuterated solvent. Efficient sample preparation has been highlighted as a necessity for NMR‐based pesticide analysis in foods (Thanou and Tsiafoulis 2024). Second, using the highest feasible magnetic field strength improves sensitivity and resolution. A stronger magnet (e.g., 600 MHz vs. 400 MHz proton frequency) increases signal strength proportionally. In fact, a 500 MHz spectrometer equipped with modern enhancements can achieve sensitivity comparable to a conventional 900 MHz system (Anklin 2015). Third, advanced hardware such as cryogenic probes (cryoprobes) is invaluable for trace analysis. Cryoprobes cool the receiver coil and pre‐amplifier to very low temperatures, dramatically reducing electronic noise. This yields a significant jump in signal‐to‐noise ratio, on the order of two‐ to fivefold sensitivity improvement over a room‐temperature probe. Such an improvement can mean detecting a contaminant at half or a quarter of the concentration previously needed. Cryogenic ^1^H–^13^C dual probes have enabled direct detection of minor adulterants and environmental pollutants in foods that would be near‐impossible to see otherwise. For example, using a cryoprobe, researchers could observe trace levels of melamine in adulterated milk powder in the proton NMR spectrum, whereas a traditional probe failed to discern it. Dynamic nuclear polarization (DNP) is another emerging technique (though not yet routine for foods) that can enhance NMR sensitivity by transferring polarization from electrons to nuclei, potentially boosting signals by orders of magnitude for low‐concentration analytes (Wolff et al. 2024). Finally, software and methodological advances help push detection limits. Techniques like signal denoising (e.g., machine learning‐assisted noise reduction) and signal averaging with intelligent stop criteria can pull out signals buried in noise. For instance, the Spectral Wavelet Analysis and Classification‐Assisted Denoising method (SWAN‐CAD) has been used to enhance weak spectral features, enabling identification of contaminants at lower concentrations. Additionally, nuclear relaxation filters can suppress overwhelming signals (like water or fat) to reveal underlying minor components. This strategy is useful in, say, detecting a polar contaminant in a lipid‐rich sample: by using T2 filters to nullify fat signals, the contaminant's signals emerge more clearly (Sobolev et al. 2022). While NMR and MRI‐based methods for food safety are sometimes limited by sensitivity, ongoing improvements in sample preparation, hardware (high‐field magnets and cryoprobes), and analytical techniques are continually extending their reach.

## 5 Applications of MR in Food Science

**FIGURE 7:**
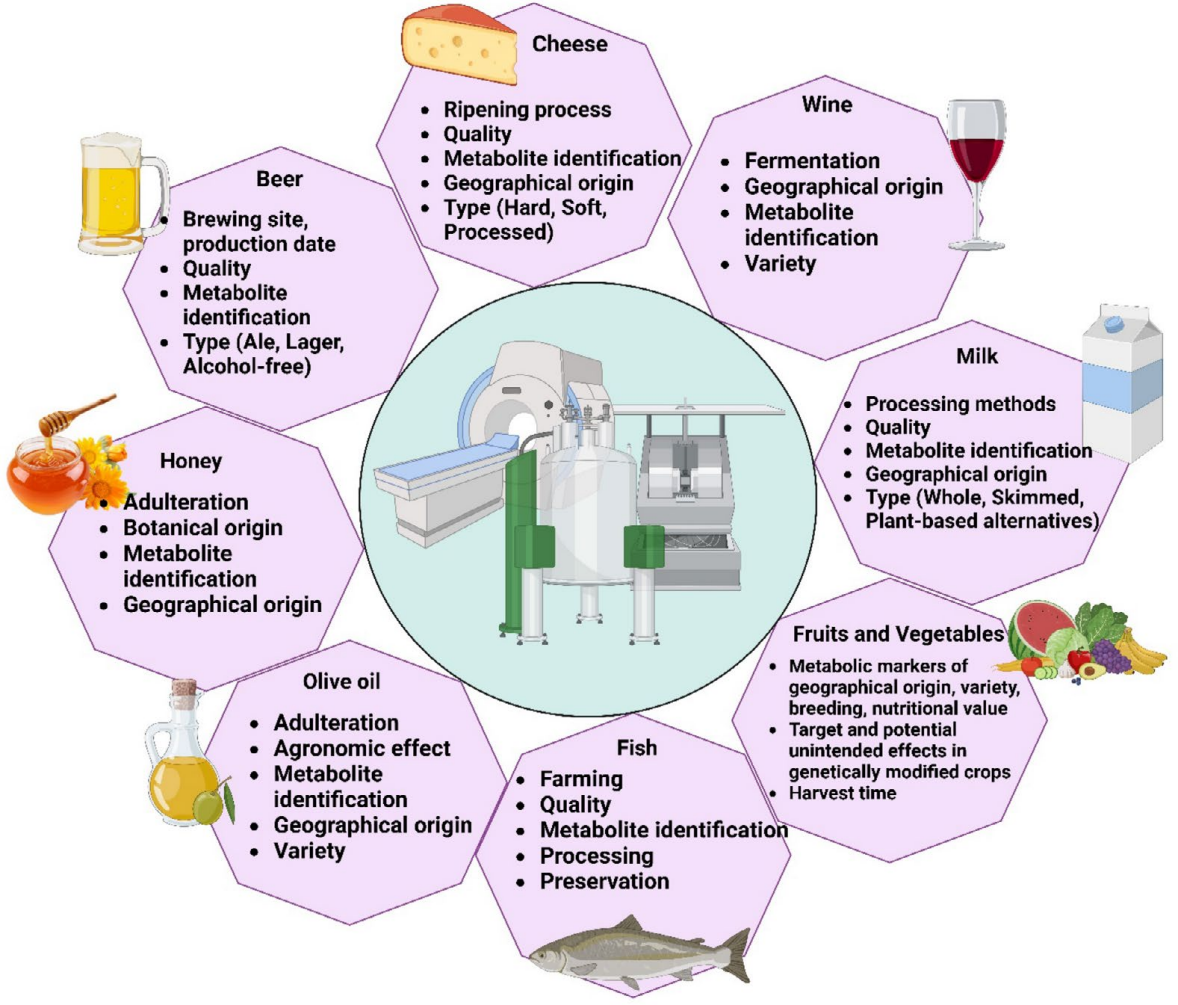
Applications of magnetic resonance (MR) in food analysis: From quality assessment to metabolite identification across diverse food products.

Magnetic resonance (MR) technologies are very important in the food science sector as they offer unique, precise, and non‐destructive methods to check up on products. The exact composition of a food, its safety, and genuineness can be found out with the help of these technologies, which include nuclear magnetic resonance (NMR), magnetic resonance imaging (MRI), electron spin resonance (ESR), magnetic resonance spectroscopy (MRS), and low‐field NMR. They are equipped with the techniques that can command food manufacturing at a continuous level, detecting adulterants, and monitoring the effect of the process. These are employed in analyzing food molecules in dairy products or plant‐based items and the safety assessment of oils and meat products. Figure 7 gives an example of the application of MR in dairy, oils, meats, and beverages for quality control and authenticity verification.

### 5.1 Food Analysis

Magnetic resonance (MR) technologies, particularly nuclear magnetic resonance (NMR) spectroscopy, are at the forefront of molecular profiling and nutritional analysis in food science. NMR allows for precise identification and quantification of various chemical components in food, including proteins, lipids, carbohydrates, and bioactive compounds. This is very important to the thing that has been considered; that is, food and nutrition (Zaukuu et al. 2022). An essential advantage of NMR in molecular profiling is that it does not disturb the original state of food products and, at the same time, it gives a comprehensive view of their molecular structure. NMR‐based metabolomics is the cutting‐edge analytical spectroscopy for the quality tracking of food matrices and their components through the entire production life cycle, right from raw product to digestive processing. This use makes the presentation of nutritional profiles clearer and the arrangement of stricter consumer demands for nutritious, high‐grade food (Qu and Jin 2022). NMR is an indispensable instrument in estimating consumption of functional ingredients that include vitamins, prebiotics, and even antioxidants in food. These substances play a very important role in the human body, involved in health maintenance, and proper determination of their presence is thus an important step in the chemical construction of functional foods. A study by (Wang, Sun, et al. 2020; Wang, Lim, and Fu 2020) investigated antioxidant mechanisms of walnut‐derived pentapeptide PW5 (Pro‐Pro‐Lys‐Asn‐Trp) were investigated using NMR spectroscopy and the ABTS radical scavenging assay. PW5 had excellent antioxidant properties with an IC50 value of 0.2210 ± 0.0032 mM and was more effective than glutathione (IC50 = 0.2567 ± 0.0023 mM; *p* < 0.001). Structural analysis indicated that the tryptophan was the primary residue to be involved, which upon oxidation was subjected to severe changes. Additionally, a study by Wiese et al. (2013) performed a microplate antioxidant assay and HPLC‐SPE‐NMR to study food extracts. Optimization of trapping efficiency, elution behavior, and recovery using solid‐phase extraction (SPE) cartridges allowed the assay to screen several common food phenolics. The best RP C18HD resins for phenolic compound entrapment were SH and GP. A proof‐of‐concept study on caper bud extract identified quercetin, kaempferol, rutin, and spermidine amides as antioxidants. Everything but NMR analysis was built up to describe these (ET) new caffeoyl‐ and coumaroyl‐substituted spermidine derivatives. It demonstrated antioxidant profiling in complicated dietary matrices. In another study, spectrophotometric methods were developed to evaluate EVOO quality and authenticity and identify adulterations. Using near UV–Vis absorption spectroscopy, ^1^H and ^13^C NMR, bioactive compounds such as pigments, secoiridoids, and squalene were analyzed in Frantoio cultivar EVOO from Tuscany, Italy. Pigmentation index, organoleptic characteristics, total phenolic content, and lipid fractions were examined (Vicario et al. 2020; Wang, Zhou, et al. 2024). Wang, Wen, et al. (2024); Wang, Zhou, et al. (2024) examined Artemisia argyi leaf flavonoids (AALFs) for antioxidant and antibacterial activities. Chemical studies indicated AALFs contain 90.03% flavonoids, 6.84% polyphenols, 1.89% proteins, and 1.24% carbs. Structural characterization employing UV–VIS, FT‐IR, and NMR confirmed typical flavonoid units. Antioxidant tests showed high radical scavenging action, with IC50 values of 0.176 mg/mL (DPPH), 0.538 mg/mL (ABTS), 0.236 mg/mL (hydroxyl), and 0.192 mg/mL (superoxide radical) with MICs of 0.8 and 0.65 mg/mL, *Escherichia coli* and *Staphylococcus aureus* were inhibited in antibacterial testing. These findings suggest AALFs could prevent food deterioration and microbiological contamination as natural preservatives. Additionally, for detects vitamins in the study by Bourafai‐Aziez et al. (2022) utilized NMR spectroscopy to analyze the metabolomic profile of acerola ( *Malpighia emarginata* D.C.) juice and concentrate powder, identifying 36 metabolites. The ascorbic acid (AA) to choline ratio and NMR profiles were proposed as potential authentication markers. Additionally, a rapid (8 min), non‐destructive ^1^H‐NMR method was developed for quantifying ascorbic acid, with a LOD of 0.05 mg/mL and LOQ of 0.15 mg/mL. These approaches provide accurate, efficient quality control tools for assessing acerola‐derived ingredients in food supplements. Moreover, the study (Park et al. 2024) was responsible for the ‘^31^P phosphorus‐31 qNMR’ quantitative phosphorus‐31 nuclear magnetic resonance (NMR) spectrum and the simultaneous determination of active vitamins B1, B2, and B6 in the multivitamin supplements. The mentioned method indicated a high specificity, linearity (*R* ^2^ > 0.999), and accuracy (99.2%–102.0%), LOD (0.04–0.07 mg/mL), and LOQ (0.14–0.24 mg/mL) for supplement analysis. Precision was ensured with the help of repeatability (1.06%–1.89%) and intermediate precision (2.51%–3.25%). In addition, the commercial supplements were successfully put into practice and proved the ^31^P qNMR method to be an effective way of rapid and practical multivitamin quality control and analysis. For probiotics, the study by Anbe‐Kitada et al. (2024) developed an internal‐calibration quantitative NMR (qNMR) method that is rapid and accurate in the measurement of rebaudioside A in probiotic‐fermented milk and other beverages. In this method, instead of liquid chromatography that is conventional in which complex sample pretreatment, a simplen is practiced by a simple dilution and solid‐phase extraction steps. The qNMR research shows a very good recovery rate (85.4%–104%) and a low relative standard deviation (1.57%–4.09%), which meets the recommendations of the international organizations. The method in question encompassed a working range of 0.80–26.7 mg/100 g with an analysis time equal to 10–20 min. In the other study by Balthazar, Guimarães, Rocha, et al. (2021); Balthazar, Guimarães, Silva, et al. (2021) a ^1^H NMR study examined the effects of *Lacticaseibacillus casei* as a probiotic culture on Minas Frescal cheese's volatile chemical components and metabolic profile. Probiotic cheese had less benzoic acid, fatty acids, volatile alcohols, and esters and more tryptophan and volatile acids than control cheese. No significant changes were observed in α‐lactose, β‐lactose, or citric acid levels. NMR and chemometrics successfully separated probiotic cheese from normal cheese, revealing that probiotic cultures accelerate proteolysis, diminish lipolysis, and change volatile component profiles. By identifying diet‐linked metabolites, NMR helps distinguish and compare vegan, vegetarian, and omnivorous diets. This analysis demonstrates the diets of people and how it affects the gut flora and metabolic health of an individual (Bertram 2022). NMR spectroscopy is widely used to analyze intricate food matrices, to detect and quantify metabolites, and identify structural elements. High‐resolution NMR has proven effective for evaluating the molecular interactions within food systems and characterizing complex compounds such as polysaccharides. NMR spectroscopy has been extensively employed in the characterization of fruits and vegetables (Dar et al. 2020). All fruits and vegetables possess distinct components that are intricately linked to their nutritional value, scent, flavor, and biological activity. For example, ^1^H NMR and 2D NMR spectroscopy were used to study pomegranates from different Iranian locales to improve their ecotype (Tang and Hatzakis 2020). The main components of pomegranate juice were glucose, sucrose, and glucoside. Citric and malic acids can distinguish ecosystems, possibly linked to climate and geography. Pakistani, Fordoss, and Kashma pomegranate juices have similar amino acid and sugar profiles, but different total phenolic content. Phenolic compounds distinguish three production regions (Hasanpour et al. 2019). Amino acids, the main metabolites of cells, can identify fruit botanical origins. Botoran's team found 12 amino acids that varied greatly across juice varietals, save glutamic acid. Stoichiometric methods (PCA and LDA) can classify plant juices by amino acid profile Amino acids, the main metabolites of cells, can identify fruit botanical origins. Botoran's team found 12 amino acids that varied greatly across juice varietals, save glutamic acid. Stoichiometric methods (PCA and LDA) can classify plant juices by amino acid profile amino acids, the main metabolites of cells, can identify fruit botanical origins. Botoran's team found 12 amino acids that varied greatly across juice varietals, save glutamic acid. Stoichiometric methods (PCA and LDA) can classify plant juices by amino acid profile (Botoran et al. 2019). In contrast, in the line water activity, the high water content of fruits and vegetables affects metabolism and growth. Water dynamics measurements reveal fruit and vegetable growth, storage, and decomposition at the microscopic level. Lv created a microwave vacuum drying (MVD) system using low‐field NMR to measure vegetable moisture. The amplitude of signal T2 shows water status. Water distribution varied by vegetable, with free water being the most lost during MVD. The signal amplitudes of bonded, fixed, free, and total water (A2) at different drying stages are strongly related to water content; however, only A2 may be used by a linear fitting model to calculate drying completion time (Lv et al. 2018). LF‐NMR spectroscopy monitored fruit and vegetable water changes during drying. Drying reduced water activity and shifted the T2 peak to the left. Both simple dried and sugar‐treated samples had decreasing T2 values, possibly due to sugar state changes during drying to reduce shrinkage. T2 relaxation length rises in salt‐treated samples due to tissue injury. Water activity was reduced with sugar and salt, although T2 relaxation length was significantly different (Chitrakar et al. 2020). Francini tested dried and fresh apples for antioxidants and polyphenols. The six fresh apple varieties have different polyphenol concentrations. Dehydration reduced oxygen radical absorption but preserved antioxidant activity. The study found a substantial association between phenol concentration and antioxidant activity (0.842, *p* < 0.001) Francini tested dried and fresh apples for antioxidants and polyphenols. The six fresh apple varieties have different polyphenol concentrations. Dehydration reduced oxygen radical absorption but preserved antioxidant activity. The study found a substantial association between phenol concentration and antioxidant activity (0.842, *p* < 0.001) Francini tested dried and fresh apples for antioxidants and polyphenols. The six fresh apple varieties have different polyphenol concentrations. Dehydration reduced oxygen radical absorption but preserved antioxidant activity. The study found a substantial association between phenol concentration and antioxidant activity (0.842, *p* < 0.001; Francini et al. 2017). On the other hand, for ESR identification of irradiated unpaired electrons in free radicals in cellulose and crystalline sugars from plant‐based meals can be used to identify irradiated fruits and vegetables (Lee et al. 2012). Stability of radiation‐induced free radicals is essential for their use as markers. The amorphous sugar and high moisture content of fresh fruits and vegetables make free radicals unstable (Guzik et al. 2008). Isolating the stiff components of irradiated fruits and vegetables, such as the skin, seeds, and shell, yields distinct ESR spectra for detection (Khan and Shahid 2022). High moisture levels affect ESR signal specificity. Hence, ESR analysis material is freeze‐dried or otherwise prepared (Polash et al. 2023). Kwon et al. used ESR spectroscopy to study radiation‐induced indicators in dried cabbage, carrot, chunggyungchae, garlic, onion, and green onion. Even 6 months after storage, ESR spectra identified radiation‐specific cellular radicals. Radiation treatment produced two cellulose radical‐related ESR peaks in addition to the principal signal (Joong‐Ho et al. 2006). After freeze‐drying oranges, grapefruits, mandarins, limes, and pineapple, Jo et al. found radiation‐induced cellulosic radicals used effectively. Some researchers found the European ESR protocol's ESR signal parameters unsatisfactory (Jo et al. 2016). Moisture, sample powder fineness (if applicable), and sample filling factor affect ESR signal saturation (Azariasl and Yasuda 2024). This observation clearly shows that the CEN standards' fixed microwave power value (0.4–0.8 mW) must be determined sample‐by‐sample. The sample's physicochemical parameters, including moisture content and temperature during irradiation, irradiation dosage, and storage conditions, affect the accuracy of a radiation‐specific cellulosic ESR signal (Sezer et al. 2023). ESR‐based irradiation history characterization does not require moisture removal from low‐moisture food samples (Fukui et al. 2023). Fresh fruits and vegetables require sample pre‐treatments to reduce moisture without affecting radiation‐induced cellulose radicals. Freeze‐drying, alcoholic extraction, oven‐drying, and various procedures can reduce moisture (González‐Pérez et al. 2023). Using fine powder from lyophilized mango pulp, Kikuchi et al. found radiation‐specific ESR in irradiated fresh mangoes after 1 week of storage. Kikuchi et al. developed another effective method for identifying irradiated fresh papaya using ESR spectra at 77 K liquid nitrogen. Solvent extraction, particularly alcoholic extraction, may be an efficient and cost‐effective way to reduce high moisture content (Kikuchi et al. 2010). Similarly, Delincée and Soika found that strawberry, papaya, and spice cellulose radicals increased ESR signals. Compared to other moisture reduction methods, alcoholic extraction worked well for food samples (Kwak et al. 2014). Irradiation of liquid sauce samples was washed with water before alcohol extraction by Akram et al. (2012). It was found that water washing and alcoholic extraction improved cellulose radical signal detection compared to freeze‐drying and alcoholic extraction. Ahn et al. found that water washing and alcoholic extraction pre‐treatment boost radiation‐induced cellulose radical ESR signals in turmeric, oregano, and cinnamon. Twenty‐one fruits, including strawberry, mulberry, lemon, banana, and others, were tested for DPPH radical scavenging antioxidant capability (Wu et al. 2024). The fruits' antioxidant capacity was measured using Vitamin C equivalent antioxidant capacity. The results were milligrams of ascorbic acid equivalent per 100 g of fruit. Each fruit was divided into fresh, immediately analyzed fruit and frozen, ground in liquid nitrogen fruit. Frozen and fresh fruits have similar VCEAC values for antioxidant capacity. The antioxidant capabilities of frozen fruits were evaluated using ESR and UV–vis spectrometry. UV–vis and ESR VCEAC values range from 11.48 to 345.75 mg/100 g and 7.01–366.26 mg/100 g, respectively. The two methodologies' VCEAC values correlated well in the experiments (Zang et al. 2017). On the other hand, for dairy products used NMR, the chemical constituents of milk powder products include lactose, protein, minerals, lipids, and vitamins A, B, and D. TD‐NMR and stoichiometry were employed to ascertain the fat content of various milk powder samples without the use of solvent (Nascimento, Barsanelli, et al. 2017). The multivariate model of T2 decay indicated that the fat percentage in solid milk powder ranged from 0.75% to 26%, while the moisture content ranged from 1.9% to 3.9%. Sanchez et al. performed a thorough characterization of whole fat goat milk powder (GMP), detecting 44 metabolites from lipid, carbohydrate, and aromatic areas via NMR analysis. The primary signals of aliphatic (0.5–3.5 ppm) and aromatic (6.0–9.5 ppm) groups are associated with metabolites including creatine, carnitine, lactose, galactose, lecithin, and sucrose. The metabolites linked to weak signals, including lactic acid and equuric acid, exhibited a substantial correlation with breast infection. These metabolites are crucial for determining GMP quality and attributes (Sanchez et al. 2020). It was employed ^1^H NMR to analyze the water‐soluble metabolomics of Alpine Asiago cheese at three maturation intervals. Lactic acids were the primary contributors to the signals in the ^1^H NMR spectra, with other prominent signals such as acetic acid, glycerol, hydrophobic amino acids, ethanol, and citric acid. 2,3‐Butanediol is a characteristic component of ranch cheese, as its concentration diminishes with aging. Lysine is correlated with a medium to short maturation duration of cheese, while phenylalanine serves as a primary signal of long‐aged cheese. The findings indicated that ^1^H NMR was an efficient technique for identifying cheese samples with the briefest ripening duration (Segato et al. 2019). Another study used bi‐exponential signal decay analysis to detect heat‐treated and raw milk cheeses using MRI. There were 36 samples: 18 heat‐treated Pecorino and 18 raw Fiore Sardo. MRI showed considerable transverse (T2) relaxation time fluctuations. Cheese made from heat‐treated milk had a greater proportion of fast‐relaxing water protons (T2 = 9 ms; 70%–80%) compared to raw milk cheeses, which had more long‐relaxing protons (T2 = 35 ms; Mulas et al. 2013). That MRI protocol is rapid, non‐invasive, and statistically distinguishes cheeses by milk treatment. MRI also tracked structural and textural changes in four Spanish sheep cheeses throughout ripening. It analyzed apparent diffusion coefficient maps and T1 and T2 relaxation durations. Additionally, cheese forms had proton density. MRIs revealed significant cheese matrix structural information about manufacturing methods (industrial vs. traditional), ripening (2–180 days), and provenance. RT and cheese variety greatly altered physicochemical and textural qualities. A lot of research supported linear regression models. Logarithmic regression models had the highest R2 values for T1 and T2 parameters, water content, activity, RT, and texture. These findings suggest that MRI can detect ripening, predict textural and physicochemical qualities, and describe sheep cheese structure (Kharbanda et al. 2023). Moreover, six materials treated (0–4 kGy) with an electron accelerator were analyzed for free radical generation, accumulation, and decay using ESR spectroscopy. Interestingly, ESR spectra of all untreated cheeses showed a singlet signal with a g‐factor of 2.0064 ± 0.0005. Interestingly, irradiated materials' ESR spectra revealed a novel signal with a g‐factor of 2.0037 ± 0.0003. This signal was consistent between cheeses and may be protein radiolysis free radicals. Surface regression models (*p* < 0.0001) revealed a correlation between signal intensity, absorbed dose (0–4 kGy), and storage time (0–180 days) in several cheeses. ESR (or electron paramagnetic resonance, EPR) can accurately measure the qualitative and quantitative effects of irradiation on cheeses (Escudero et al. 2019). In addition, Electron spin resonance (ESR) spectroscopy and spin‐trapping can examine dietary lipids' oxidative stability, focusing on radical generation. Assessment of induction time under modestly accelerated circumstances (50°C for fish oil‐enriched mayonnaise lipid fraction) shows that it shortens with storage and γ‐tocopherol depletion (Jiang et al. 2020). The novel assay shows that ethylene diamine tetra acetic acid prevents lipid oxidation. If the difference in signal height at fixed times is employed instead of the induction time, the test can be mildly expedited for more oxidatively stable lipids like butter, rapeseed oil, and dairy spread. ESR spin trapping accurately measures food lipid oxidative stability. The identification of radicals in lipids as a first event in oxidation allows moderate settings, and subsequent tests should include sensory assessment of shelf life. Table 1 shows a summary of the application MR for food analysis. Finally, magnetic resonance (MR) techniques (NMR, MRI, and ESR) offer a uniquely comprehensive and non‐destructive approach to food composition analysis. MR tools can profile intact food samples with virtually no preparation or separation, so the food matrix remains unaltered by the assay. In one NMR experiment, hundreds of components (metabolites, lipids, sugars, etc.) can be quantified simultaneously, yielding a rich chemical fingerprint (Gowda and Raftery 2015). MRI further adds real‐time structural insight by imaging internal distributions of water, fat, and other constituents without slicing the sample. Likewise, ESR provides a non‐destructive, highly sensitive probe of free radicals and other paramagnetic species in food. These features—high reproducibility, multi‐analyte detection, and minimal prep—make MR methods exceptionally powerful for detailed compositional and metabolomic profiling of complex food matrices (Mohammadi‐Moghaddam et al. 2024; Watanabe et al. 2023).

**TABLE 1:** Application magnetic resonance in food analysis.

Type of MR	Type of food	Aim	References
MRI	Dry‐cured ham	Characterizing tissues and structural changes during processing	Bajd et al. (2016)
MRI	Dry‐cured loins	Studying sensory attributes using various MRI acquisition sequences and analysis algorithms	Caballero et al. (2017)
MRI	Chicken breast meat	Assessing effects of repetitive freeze/thaw cycles on quality attributes	Frelka et al. (2018)
MRI	Salmon steaks	Measuring mass fat fraction in a high‐throughput manner	Picaud et al. (2016)
LF‐NMR and MRI	Broccoli and mushrooms	Monitoring hot‐air drying processes and water distribution	Xu et al. (2017)
LF‐NMR and MRI	Prawns	Detecting hydrocolloid adulteration	Li, Li, and Zhang (2018)
Low‐field MRI	Live crabs	Quantifying lipid tissue for grading and breeding advice	Zhang et al. (2018)
MRI	Turbo flesh	Analyzing water dynamics during frying, boiling, and stewing	Xia et al. (2018)
T2 Relaxometry and MRI	Fresh noodles	Studying storage‐induced changes and polyol effects on noodle texture	Li, Wang, et al. (2018)
MRI	Fruits	Monitoring ripening and postharvest quality	Panebianco et al. (2024)
MRI	Meat	Analyzing effects of freeze–thaw cycles on quality	Caballero et al. (2021)
MRI	Fried potatoes	Investigating oil and moisture distribution in fried foods	Yang et al. (2020)
LF‐NMR and MRI	Frozen‐cooked noodles	Studying texture and quality changes during storage and processing	Roh et al. (2020)
^1^H NMR	Swabian–Hall Quality Pork	Studying to identified to describe potential chemical marker compounds	Decker et al. (2023)
^1^H NMR	Meat	Classify the beef samples by breed, cattle type, aging time, and aging type	Bischof et al. (2023)
^1^H NMR	Beef and horse meat	Distinguish beef from horse meat based on triglyceride profiles using Naïve Bayes and PCA	Jakes et al. (2015)
^1^H NMR	Iberian dry‐cured hams	Developing fatty acid profiles and assessing nutritional and sensory contributions	Pajuelo et al. (2023)
^1^H NMR with 2D NMR	Dry‐cured ham	Characterizing flavor‐related metabolites and correlating them with sensory attributes	Pajuelo et al. (2022)
^1^H, ^13^C, DEPT‐135 NMR	Fish oil	Evaluating a solvent‐free extraction technique and comparing fatty acid profiles	Chakraborty and Joseph (2015)
Low‐field (LF) NMR	Avocado oil	Quantify fatty acid composition (poly‐, mono‐, saturated) and authenticate avocado oil vs. other oils	Tang et al. (2024)
Ultrafast 2D NMR (43 MHz benchtop)	Edible oils (olive, hazelnut, sesame, rapeseed, corn, sunflower)	Classify oils of different botanical origins and detect adulteration (e.g., hazelnut in olive oil)	Gouilleux et al. (2018)
Benchtop ^1^H NMR (60 MHz)	Olive oil adulterated with hazelnut oil	Detect and quantify hazelnut oil adulteration in olive oil; compare with FTIR for sensitivity/specificity	Parker et al. (2014)
^1^H NMR	Cheese	Profiling of cheese triacylglycerols can identify specific biomarkers for cheese classification according to producing species, origin, and variety	Haddad et al. (2022)
^1^H NMR	Grapevine and wine	Analytical techniques to characterize wine composition and origin	Bambina et al. (2024)
^1^H NMR with 2D NMR	Coffee beans	Identifying metabolites linked to quality and grade, and effects of roast levels	Ripper et al. (2020)
^1^H NMR with 2D NMR	Cherry tomatoes	Metabolomics profiling in the geographical determination of food origin	Masetti et al. (2020)
^1^H NMR with 2D NMR	Zucchini	Evaluating the effects of irrigation and ventilation techniques on crop yield and quality	Abreu et al. (2018)
^1^H NMR	Sweet cherries	Monitoring qualitative changes during storage using metabolic fingerprinting	Longobardi et al. (2013)
^1^H NMR	Grape juice	Differentiating production methods and storage effects based on chemical fingerprints	Grandizoli et al. (2014)
^31^P NMR	Chocolate	Quantifying ammonium phosphatide emulsifiers and correlating with quality attributes	Malmos et al. (2018)
^1^H NMR with OPLS	Extra virgin olive oil	Establishing sensory parameter correlations with metabolic profiles using NMR fingerprinting	Ruiz‐Aracama et al. (2017)
^1^H NMR with PCA	Honey	Identifying botanical origin and biomarkers for specific varieties	Zheng et al. (2016)
Conventional MRI	Dry‐cured ham	Characterizing structural changes during different stages of curing	Fantazzini et al. (2009)
Conventional MRI	Wine grapes	Differentiating fertilization effects and predicting grape split	Mian et al. (2024)
Conventional MRI	Fruits	Monitoring ripening and postharvest quality	Fatemi et al. (2022)
Conventional MRI	Meat	Analyzing effects of freeze–thaw cycles on quality	Liu et al. (2022)
CW X‐band EPR	Scotch whisky	Detection of Fe^3+^, Mn^2+^, and Cu^2+^ metal ions to study origin and degradation processes	Zoleo et al. (2015)
CW ESR (X‐band)	Mushrooms	Determining free radicals in pigmented and unpigmented varieties	Bercu et al. (2011)
CW ESR with imaging	Sesame seeds	Identifying radiation‐induced radicals in toasted and irradiated seeds	Nakagawa and Hara (2015)
CW ESR	Dry herbs and spices	Detecting free radicals to verify irradiation for microbial control	Jin, Zhang, et al. (2020)
X‐band ESR	Coffee beans	Analyzing organic radicals formed during roasting (Maillard reaction)	Ece et al. (2018)
CW ESR with spin trap	Olive oil	Investigating lipid oxidation and radical generation during heating	Rahmani‐Manglano et al. (2023)
CW ESR with imaging	Shiitake mushrooms	Mapping the spatial distribution of radical species in dried and fresh mushrooms	Nakagawa and Hara (2016)
CW ESR (L‐band)	Plant seeds	Monitoring paramagnetic species and oxidative processes in irradiated seeds	Khan and Shahid (2022)
CW ESR	Cheese	Measuring antioxidant properties and assessing radical scavenging capacity	Escudero et al. (2019)
X‐band ESR	Dark beer	Characterizing stable organic radicals in beverages and their relation to flavor	Hrabia et al. (2022)
High‐field ESR	Crystalline sugar	Identifying radicals induced by gamma irradiation for food safety verification	Kimura et al. (2021)
CW ESR	Irradiated meats	Detection of free radicals formed by ionizing radiation for authenticity verification	Vazirov et al. (2024)
CW ESR with spin probe	Bottled beer	Analyzing antioxidant properties by trapping reactive oxygen species (ROS)	Dobosz et al. (2022)
CW ESR	Tea leaves	Correlating semiquinone radicals with flavonoid and antioxidant content	Mwachiro et al. (2019)
CW ESR	Irradiated poultry	Monitoring stability and concentration of radicals induced by gamma sterilization	Lea et al. (2007)
CW ESR (X‐band)	Green vegetables	Studying the effect of UV light on radical formation and oxidative stability	Kwak et al. (2014)

### 5.2 Food Safety and Contaminant Detection

Magnetic resonance (MR) technologies, including nuclear magnetic resonance (NMR), magnetic resonance imaging (MRI), electron spin resonance (ESR), magnetic resonance spectroscopy (MRS), and low‐field NMR, are transformative tools in food science, particularly for food safety and contaminant detection. These technologies offer unparalleled capabilities to analyze molecular interactions, identify contaminants, and assess quality parameters in food products. For instance, low‐field NMR has been deployed for cost‐effective microbial detection, exemplified by the development of a low‐field MRI‐based aptasensor. This technology detects *Pseudomonas aeruginosa* in foods and beverages with a sensitivity of 100 CFU/mL, providing rapid, quantitative results suitable for practical applications in various food matrices (Jia et al. 2021). In addition to detecting hazards, MR‐supported approaches are helping elucidate how foodborne pathogens adapt and survive under stress, which is crucial for food safety management. Nuclear Magnetic Resonance (NMR)‐mediated metabolomics coupled with advanced omics tools allow us to see the molecular adaptations of the foodborne pathogen *Salmonella*. For example, the revelation of the transcriptome of *Salmonella enterica* by genome sequencing technology has been intimately linked to gene expression changes in response to food processing and preservation conditions. Under oxidative disinfectant stress (e.g., sodium hypochlorite), *S. enteritidis* was found to differentially express over 1300 genes; notably, NMR‐assisted metabolite profiling and RNA sequencing showed upregulation of membrane proteins and efflux pumps that help expel the sanitizer, as well as shifts in energy metabolism that push cells toward a dormant, tolerant state (Wang et al. 2022). Likewise, during acid stress adaptation, *Salmonella* upregulates genes linked to virulence, stress resistance (toxicant‐antitoxin systems), and iron acquisition (Ghoshal et al. 2024). These transcriptomic insights indicate that sublethal acid exposure, such as in acidic foods or cleaning agents, can induce more robust, potentially more virulent phenotypes in the pathogen. By integrating NMR‐based metabolomics with transcriptomics, researchers can correlate gene expression changes with metabolic shifts (e.g., accumulation of stress‐protective metabolites), yielding a comprehensive understanding of pathogen physiology under stress (Chen et al. 2022). Such integrative MR‐omics studies are invaluable: they highlight targets for intervention (e.g., inhibiting efflux pump activity or acid tolerance pathways) and inform the design of more effective control strategies to curb *Salmonella* and other pathogens in the food chain. In addition, a nuclear magnetic resonance biosensor using a streptavidin‐biotin system has demonstrated success in detecting *Salmonella* in milk. This biosensor achieves rapid detection within 1.5 h and offers high sensitivity (103 CFU/mL) while maintaining robustness in complex matrices, highlighting its potential as a nondestructive food safety tool (Dong et al. 2022). Moreover, bacterial biofilms represent another major challenge in food safety, as they protect microbes from sanitizers and can persist on food contact surfaces. NMR‐supported metabolite profiling has emerged as a powerful approach to study biofilm metabolism and identify ways to disrupt these resilient communities. For example, recent work employed ^1^H NMR‐based metabolomics to investigate the responses of *Escherichia coli* biofilms to combined sanitization treatments (acidic electrolyzed water and ultrasound). By analyzing the biofilm's metabolite profiles, researchers found that ultrasound primarily disrupted nucleotide metabolism (e.g., significant depletion of ATP, ADP, and other nucleotides), while mild acid oxidants affected amino acid and carbohydrate levels in the biofilm cells (Zhao et al. 2022). Under the combined treatment, *E. coli* biofilms activated adaptive stress responses such as the glutamate decarboxylase acid resistance system and shifted to fermentative pathways. These metabolic signatures, captured via NMR, revealed how biofilm cells attempt to survive sanitization. Importantly, the same study demonstrated that NMR‐based metabolomics can discern strain‐specific metabolic changes in biofilms, meaning different strains of a species may utilize distinct protective mechanisms (Chen et al. 2020). On the other hand, moisture state and microbiota makeup, food water content and physical state affect microbial growth, community makeup, and spoilage rate. Low‐field NMR (LF‐NMR) is effective for monitoring food moisture and microbiological stability. LF‐NMR relaxometry can identify bound water (tightly bound to food matrices) from free or mobile water and follow their changes throughout storage or processing. NMR relaxation times (T1) are linked to freshness, texture, and microbiological deterioration markers. In chilled salmon fillets, LF‐NMR studies indicated that as storage time rose, the proportion of trapped (bound) water declined and free water increased, matching with growing spoiling metabolites (total volatile bases, etc.) and poor sensory ratings. This suggests microorganisms and chemical processes are gaining access to water, increasing deterioration. NMR investigations have demonstrated considerable relationships between moisture mobility and spoiling processes: samples with more bound water had slower microbial growth and lipid oxidation, whereas samples with more free water had better spoilage organism proliferation. LF‐NMR may predict microbiota activity by non‐invasively monitoring water distribution. A larger free water percentage generally precedes microbial bloom and spoiling microbiota community alterations. In one research, LF‐NMR could distinguish the effects of storage temperature on fish muscle quality, showing how water qualities affect bacterial deterioration. Water dynamics inform food preservation since limiting water activity (drying, salting, etc.) inhibits microbial development and may be monitored by NMR. Researchers have even incorporated LF‐NMR into drying equipment to monitor fruit/vegetable moisture reduction in real time and decide when a safe low water activity is attained. LF‐NMR shows food water at a microscopic level and throughout time. This information is significant since moisture status affects microbial composition, food safety, and shelf life. Producers can regulate microbiological stability and spoilage by knowing and managing water mobility (e.g., packing, humectants, and drying procedures). NMR can evaluate and improve these interventions (Wang et al. 2018). In addition to pathogen detection, MR technologies have advanced the monitoring of toxin responses in living organisms. Using the time‐resolved NMR method, researchers were able to identify sub‐lethal concentrations of the toxin bisphenol A (BPA), the uptake of which guided the exploration of metabolic pathways impacted by the toxin exposure. This is the main feature of modern toxicity research, as this model offers quicker, more organized, and, most importantly, capable analysis of contaminant effects (Lane et al. 2020). Meanwhile, low‐field NMR has become increasingly accessible for broader industrial applications. Innovations such as selective detection, heteronuclear experiments, and spectral simplification overcome the limitations of low‐field MR in analyzing heterogeneous food samples, making it a practical tool for assessing food quality in real‐world scenarios (Downey et al. 2023). Moreover, a high‐temperature superconductor (HTS) SQUID and a permanent magnet were used to create an ultra‐low field NMR/MRI device for food and beverage contamination detection. Two permanent magnets with 1.1 T fields prepolarized the HTS‐SQUID‐based device to increase the signal‐to‐noise ratio. A sample transfer device moved a 10‐mL glass bottle of dirty water from the magnet to below the SQUID magnetometer in 0.5 s. The method demonstrated beverage contaminant identification using NMR and 1‐D MRI of water samples with stainless steel, aluminum, and polymer pollutants. Filtered back projection reconstruction was used for a silicone bulk‐partitioned water sample 2‐D MRI (Hatsukade et al. 2013). More progress in Magnetic Resonance Spectroscopy (MRS) has made it possible to exactly find out the molecular differences in food matrices. Thanks to these technologies, it is now a matter of recovering the metabolic markers and contaminants present in the materials with the greatest precision. On the contrary, MR methods have always had trouble with the difficulties such as traditional low sensitivity and long acquisition times, especially for low‐concentration analytes detection. Recent advances, especially deep learning‐based denoising, have tackled the technology's shortcomings by increasing the signal‐to‐noise ratio (SNR) and decreasing acquisition times. For example, new methods such as Spectral Wavelet‐feature Analysis and Classification Assisted Denoising (SWANCAD) enhance the SNR of low NSA (number of signal averages) spectra, enabling faster scans without compromising spectroscopic information (Ji et al. 2021). Quantum MR technologies, particularly nitrogen‐vacancy (NV) center‐based quantum sensors, are paving the way for nanoscale sensitivity in food safety applications. These sensors allow the detection of contaminants and structural details with exceptional precision, presenting opportunities for applications in single‐cell studies, lab‐on‐a‐chip devices, and molecular‐level food safety assessments (Rizzato et al. 2023). Additionally, by creating portable MR devices, it becomes possible to carry out tests on‐site. Just to give an example, zero‐field NMR devices can provide an analytical performance level for small biomolecules and even complex materials, thus helping researchers and even agricultural and heavy industries to do a real‐time analysis without a high‐field lab setup (Blanchard et al. 2019).

### 5.3 Food Authentication

Magnetic resonance (MR) techniques, such as nuclear magnetic resonance spectroscopy (NMR), magnetic resonance imaging (MRI), and electron spin resonance (ESR), have now become a necessary tool in the field of food authentication. The advent of such methods has provided us with atomic resolution information. This allows for the correct discovery of the origins of the food, its composition, and the possibility of adding disguised substances. In the particular case of NMR, spectroscopy is used for analyzing food that is too complex. It has the added advantage of being non‐destructive testing and minimal sample preparation and is well classified as having high specificity. For instance, NMR has been used in wine authentication by grape variety identification with non‐targeted spectroscopy. The researchers standardized sample preparation and spectrometer protocols to avoid classification deviations across different labs. This was achieved by the researchers, and they had specificity in their tests ranging from 79% to 87% (Ragone et al. 2020). Authenticity analysis of wine making is supported by sophisticated analytical methods that can tell apart the grape varieties, origin, and age. High‐resolution NMR (nuclear magnetic resonance) has proven to be the most efficient for the detection of specific grape byproducts like shikimic acid and tartaric acid, which are markers of oxidation during wine making (Gnilomedova et al. 2023). Additionally, proline, the most abundant amino acid in wine, is often used as a marker for ripeness (Gutiérrez‐Gamboa et al. 2018). Polyphenols, especially those extracted from wine, are analyzed via proton NMR (^1^H NMR) to determine variety, origin, and vintage, as demonstrated in studies on Greek wines (Anastasiadi et al. 2009). Adulteration of wine, such as the addition of sugars before fermentation, is another common issue. Techniques like Site‐Specific Natural Isotope Fractionation by NMR (SNIF‐NMR) combined with isotope‐ratio mass spectrometry have been utilized to trace exogenous sugars derived from sources like beet and cane (Rojas‐Rioseco et al. 2024). Untargeted NMR procedures can also authenticate wine. These methods use powerful multivariate statistical algorithms to detect wine type, origin, vintage, and production processes across the NMR spectrum. Essential characteristics like anthocyanins determine red wine authenticity. They are crucial for color assessment but can also reveal deceptions like fortifying with other wines or adding anthocyanins from plants like black rice. The ^1^H NMR method and statistical analysis have been successful in identifying anthocyanin sources in unidentified wine samples. A single study classified five wines—white, rosé, and three red—using Partial Least Squares Discriminant Analysis (PLS‐DA) and the wavelet‐linear model. Wavelet‐based classification was the most successful in this situation, with a classification rate of 95% (Ferrari et al. 2011).

NMR technology is not only limited to dairy and wine but also used in spices and condiments, among other things, serving as a powerful tool of adulterant detection and a reliable method for location verification. The chemometric treatment of NMR spectra enables the exact determination of certain constituents in multifaceted mixtures, presenting a reliable technique for a large number of quality control tests (Belmonte‐Sánchez et al. 2020). Spices are increasingly analyzed using nuclear magnetic resonance (NMR) techniques due to their ability to provide detailed molecular insights. Initially, methods such as liquid CO2 extraction coupled with NMR were utilized to characterize specific compounds like anethole in fennel seeds and methyleugenol in basil (Pacholczyk‐Sienicka et al. 2021). NMR has also been employed to monitor metabolic changes in spices, detecting and quantifying both primary and secondary metabolites in aromatic Mediterranean spices traditionally used in cooking. Complex methods such as HPLC‐SPE‐NMR enable the characterization of major metabolites in extracts like sage ( *Salvia fruticosa* ), which are investigated for antifungal properties and general chemical profiling. Among spices, saffron (*Crocus sativus L*.) stands out as one of the most complex and expensive to authenticate. Saffron's quality and authenticity depend on its key compounds: safranal (flavor), picrocrocin (bitterness), and crocin (coloring intensity; Exarchou et al. n.d.). However, its high price makes it susceptible to adulteration. Common practices include mixing saffron with inferior parts of the plant, such as petals and styles, or blending it with coloring agents from unrelated plants like safflower or turmeric. Other fraudulent techniques involve using marigold leaves ( *Calendula officinalis* ), arnica ( *Arnica montana* ), or artificially colored materials to mimic saffron's appearance.

**FIGURE 8:**
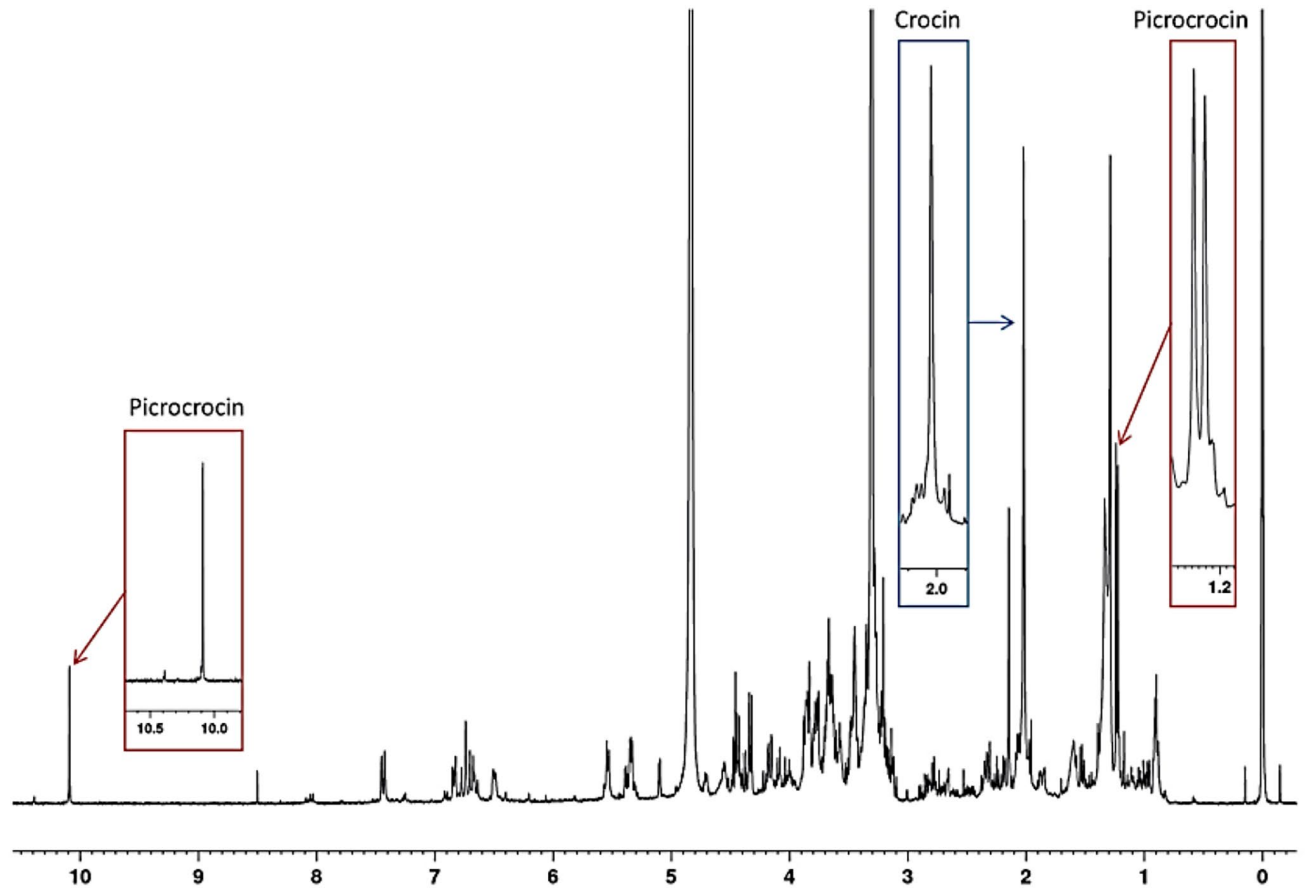
A genuine saffron sample's ^1^H NMR spectrum with picrocrocin and crocin resonances emphasized and magnified. *Source:* Schumacher et al. (Schumacher et al. 2016). No permission needed (CC BY‐NC‐ND 4.0).

^1^H NMR has proven effective in detecting saffron adulteration through a metabolite fingerprinting approach combined with chemometric methods like PLS‐DA. Studies have demonstrated the ability of NMR to distinguish pure saffron samples from fraudulent ones and assess the influence of adulteration on shelf life. A complete saffron NMR spectrum is given in Figure 8 (Schumacher et al. 2016). The evaluation of NMR signals suggests that it can discriminate between authentic and fake saffron samples. FT‐IR and NMR are a lethal combination so that saffron's properties and changes it is subjected to may also be recognized. One example of the undirected spectral fingerprinting method is incredibly important to detect the unexpected falsified products. It demonstrates the usefulness of NMR technology in securing the genuineness of saffron and other spices. However, the application of NMR in spice authentication is not developed yet, and the feasibility studies are the main theme (Musio et al. 2022). There is not any commercial product that uses NMR in the daily routine for spice authenticity check, but many believe that it would definitely be very advantageous in the future. In much the same way, quick and easy NMR relaxometry is now becoming fashionable to study water products like fish, the examination being carried out for moisture, fat, and protein. The method is quite accurate and reliable in the control of spoilage and determination of the fish supply chain integrity (Wang et al. 2021). A further advancement is the inclusion of tabletop NMR instruments that are an economical as well as user‐friendly substitute for the control of the food production process. These devices can accurately measure the chemical shifts and relaxation times, thus classifying the food components during the processing stage (Hara et al. 2022). Furthermore, NMR spectroscopy has shown potential in the authentication of nanoencapsulated food ingredients, offering detailed structural analysis and tracking molecular interactions in functional foods (Koshani and Jafari 2020). This Table 2 summarizes the applications of various Magnetic Resonance (MR) techniques, including HR NMR, ^1^H NMR, LF‐NMR, MRI, TD‐NMR, and ESR, in authenticating a wide range of food products.

**TABLE 2:** Food authentication using magnetic resonance (MR) technologies.

Type of MR	Type of food	Aim	References
HR NMR	Virgin olive oil	Authentication of geographical and varietal origin, using fatty acid profiles and PCA analysis	Kritioti et al. (2018)
	Wines	Authentication based on geographical and varietal origin using multivariate analyses	Geană et al. (2019)
	Sweet melon	Varietal classification and correlation with sensory characteristics	Girelli et al. (2018)
	Hempseed oil	Detection of adulteration	Siudem et al. (2022)
	Coffee	Authentication and detection of adulteration	Wang, Lim, and Fu (2020)
	Saffron	Authentication and identification of varietal and geographical origin markers	Raina et al. (2023)
	Green tea	Discrimination of geographical origins and understanding correlations with climatic factors	Lee et al. (2010)
	Honey	Authentication and detection of sugar syrup adulteration	Zhang and Abdulla (2022)
	Beef	Authentication of pre‐slaughter production systems and geographical origin	Bischof et al. (2023)
	Edible oils (general)	Authentication, including discrimination between types based on sterol and fatty acid content	Tang et al. (2021)
	Saffron (Italian PDO)	Identification of key metabolites (e.g., crocins) for authentication	Gunning et al. (2022)
	Lager beer	Authentication by detecting carbohydrate profile variations	da Silva et al. (2019)
^1^H NMR (High Field)	Fruits and Vegetables	Evaluate water distribution, quality, and storage impacts	Mascellani Bergo et al. (2024)
LF‐NMR	Meat and Aquatic Products	Assess water mobility, protein content, and shelf‐life quality	Gudjónsdóttir et al. (2011)
MRI	Processed Foods	Observe internal structures, moisture migration, and processing effects	Guo et al. (2024); Mayar et al. (2024)
LF‐NMR	Honey	Detect botanical origin and adulteration with sugar syrups	(Damto et al. 2024)
TD‐NMR	Fruit Juices	Authenticate origin, identify quality changes during storage	Navarro et al. (2020)
ESR	Nuts	Measure lipid oxidation for quality control	Yu and Cheng (2008)
MRI	Fish	Monitor freshness and spoilage	Sharma et al. (2023)
NMR	Soy Products	Detect adulterants and assess quality	Ethier et al. (2024)
MRI	Frozen Foods	Evaluate freeze damage and structural changes	Kind et al. (2023)
ESR	Cooking Oils	Analyze oxidative stability during frying	Marinova et al. (2012)

### 5.4 Detect Adulterated Food

Individual food elements or combinations of them might highlight a food product's attributes. These constituents' composition and behavior in complex food items provide vital insights into food science and food product development (Metilli et al. 2020). For oil, the types and arrangement of fatty acids in triacylglycerols and their linkages to the glycerol backbone identify olive oil and other vegetable oils. Olive oil's triacylglycerol is mostly oleyl and linoleyl unsaturated and palmitic and stearic saturated. Oil types affect acyl and acyl positional distribution. Olive oil quality depends on origin and manufacture. Identifying adulteration requires acyl presence and, geographic dispersion. Chromatographic methods detect other oil‐induced virgin olive oil adulteration. They have considerable drawbacks (Indelicato et al. 2017). They lack specificity, are damaging, time‐consuming, and low‐quality. Consequently, advanced techniques using high‐resolution ^1^H and ^13^C NMR spectroscopy were developed for the examination of virgin olive oil. Vlahov and Sacchi conducted a comprehensive evaluation of the primary discoveries. Adulteration of soybean, peanut, and maize seed oils, which contain n‐3 linolenic acid, can be detected through testing (Alharbi et al. 2024). ^13^C NMR is a valuable tool for the analysis of fatty acids. The presence of olefinic, methylenic, and carbonyl groups can be used to accurately determine the levels of fatty acids in olive oil. NMR analysis can differentiate between processed oils and virgin olive oils (such as “olive oils” and “olive pomace oils”) by quantifying the overall quantity of diacylglycerols and the ratio of sn‐1,3‐diacylglycerols to the total of sn‐1,2 and sn‐1,3 diacylglycerols (Dais and Hatzakis 2013). For trans fatty acid detection in virgin olive oil, NMR can replace gas chromatographic methods (Xu et al. 2014). Virgin olive oil with trans fatty acids is considered impure. However, oleyl, linoleum, and linolenic trans isomers may also be present in the refined olive and olive pomace oils. The EU uses capillary gas chromatography standards. Virgin and refined olive oils were analyzed using ^13^C NMR and GC (Maestrello et al. 2022). Virgin olive oil had no trans isomers, but processed olive oil had 0.3%–1% trans fatty acids. The purity of virgin olive oil can be assessed by analyzing its unsaponifiable matter, which mostly consists of squalene, β‐sitosterol, and aliphatic alcohols. The differentiation of virgin, olive‐pomace, and refined olive oils has been achieved through the application of multivariate statistical analysis and ^13^C NMR (Giacometti 2001). In addition to wine, for genuine wine certification, multitechnical and q‐NMR analysis were used to distinguish between authentic and fake wine. Sample B revealed variations in succinic acid, acetaldehyde, inositol, and isoamyl alcohol, while sample C showed variations in methanol, gallic acid, glucose, and threonine. Gougeon et al. found a variation in grape variety between counterfeit and legitimate wine (Gougeon et al. 2019). It was demonstrated the use of nuclear magnetic resonance (NMR) profiling to classify and quantify binary wine mixtures. In the first step, linear discriminant analysis (LDA) effectively identified the type of mixture based on NMR spectra. In the second step, a single‐layer artificial neural network was used to quantify the relative proportion of each wine in the mixture, achieving a precision of approximately 10%. This two‐step approach confirms the potential of NMR combined with multivariate analysis for accurate wine authentication and mixture analysis (Imparato et al. 2011). Kuballa and his team employed ^1^H NMR spectroscopy to conduct a comparative analysis of suspected Russian and Kenyan liquor samples with authentic ones. Russian counterfeits were adulterated with flavorings and colorants in diluted alcohol. The concentration of methanol in counterfeit vogat was 16 times higher than in the original vogat. The Kenyan sample exhibited the presence of glycerin and a minimal quantity of higher alcohol (Kuballa et al. 2018). The composition of Scotch whisky was analyzed using 1D and 2D NMR spectroscopy, and its classification was determined using Principal Component Analysis (PCA) and Orthogonal Projections to Latent Structures Discriminant Analysis (OPLS‐DA). 3‐methylbutanol was essential in accurately categorizing blend and malt whisky. During the authenticity testing, it was discovered that the counterfeit whisky had glycerol, sugar, and an excessive amount of vanillin. These chemicals serve as distinguishing factors between genuine whisky and counterfeit versions (Kew et al. 2019). Also, for honey, the rising popularity of honey due to its unique taste and health benefits has contaminated numerous syrups. We used cheap sweeteners such as refined sucrose, beet sugar, HFCS, and maltose syrup. Sugar or syrup in bee food is contaminating honey more often. Misclassification of honey by botanical or geographical origin was also detected (Guler et al. 2014). The study examined pure honey and honey with varied high fructose corn syrup concentrations. The analysis included low‐field NMR and physicochemical evaluation. Water composition, pH, water activity, ash concentration, and color can distinguish polluted nectar from pure nectar. The relaxing time of pure honey depends on its dopant concentration. As fructose syrup concentration increased, relaxation time decreased. The LF‐^1^H‐NMR may detect varied levels of adulteration (Ribeiro et al. 2014). Due to the flavor and likeness of rice syrup and honey, honey adulteration has increased. We used PCA to analyze honey and brown rice syrup (^1^H NMR spectra). In an NMR honey analysis, a characteristic peak at δ 5.39 ppm indicates the presence of brown rice syrup (BRS) adulterant. Using a ^1^H quantitative NMR approach, researchers quantified this syrup adulterant in two commercial honey samples. The method was fast and precise, with a high reproducibility (precision 0.1%–1%) and detection accuracy around 22%–37% (Musharraf et al. 2016). On the other hand, for dairy products, nuclear magnetic resonance (NMR) is a highly efficient technique for detecting the existence of contaminants in dairy products (Mafra et al. 2022). Li et al. used NMR spectroscopy to differentiate goat, cow, and soy milk and detect soy milk in milk samples. Examining 1D and 2D NMR spectra identified 11 metabolites. PCA categorization included ten metabolites: N‐acetyl carbohydrate, carnitine, choline, acetic acid, citric acid, ethanolamine, D‐sucrose, creatine, D‐lactose and lecithin. Adulteration also allows routine testing. The LOQ for milk adulteration is 2% (v/v). Soy milk adulteration detection has a 2% relative standard deviation. Goat milk has 5% adulteration (Li et al. 2017).

Santos et al. introduced a new approach to schematics for quality control in milk by using ^1^H TD‐NMR. Whey, urea, hydrogen peroxide, synthetic urine, and synthetic milk were added to the milk at volumetric concentrations of 5%–15%–25%–35% and 50%, respectively. From the studied dispersion index of the ^1^H TD‐NMR relaxation attenuation, it could be proven that the milk sample contained only water, and T2 relaxation time was found to react significantly to changes in the doping level (Santos et al. 2016). High‐field ^1^H NMR spectroscopy and conformance index analysis distinguish genuine skimmed milk powder from adulterated. The lowest concentration of nitrogen‐rich minor compounds like melamine and dicyandiamide in samples is 0.005%–0.05% (w/w). The minimum milk metabolite concentration for urea, sucrose, and maltodextrin detection was 0.5% (w/w). Bergana et al. (2019) suggested that 5% w/w milk metabolites could indicate adulteration. The aforementioned findings were consistent with the analysis of tainted milk powder conducted by Harnly and colleagues using proton NMR. This study also highlighted the efficacy of NMR technique in detecting contaminated milk powder (Harnly et al. 2018). Finally, more applications of MR for Table 3 highlight the role of magnetic resonance (MR) techniques, such as NMR spectroscopy and MRI, in detecting adulteration across various food products.

**TABLE 3:** Detection of food products adulteration by MR method.

Food product	MR method	Type of adulteration detected	References/source
Olive Oil	NMR Spectroscopy	Adulteration with cheaper oils (e.g., sunflower)	Lanza (2023)
Honey	High magnetic field NMR	Detection of sugar syrups and water addition	Rhee et al. (2023)
Milk	NMR Spectroscopy	Adulteration with water and synthetic milk	Vlasiou (2023)
Meat Products	NMR Spectroscopy	Identification of non‐meat fillers and additives	He et al. (2023)
Fruit Juices	NMR Spectroscopy	Detection of added sugars and artificial flavors	Mac et al. (2023)
Wine	NMR Spectroscopy	Detection of water, sugar, and synthetic alcohol	Mac et al. (2023)
Dairy Products	NMR Spectroscopy	Detection of plant‐based milk and non‐dairy fats	Yuan et al. (2023)

### 5.5 Solid‐State NMR Applications in Food Science

While most routine food NMR analyses are done in solution (liquid‐state NMR of extracts or liquids), many food systems are not readily amenable to solution NMR—these include solid or semi‐solid foods, gels, powders, and insoluble biopolymers. Solid‐state NMR (ssNMR) spectroscopy has therefore become an important tool to investigate foods in forms that mimic their real textures, providing insight into molecular structure and dynamics that cannot be obtained from solution studies. Solid‐state NMR employs techniques such as Magic Angle Spinning (MAS) and Cross‐Polarization (CP) to obtain high‐resolution spectra of rigid or heterogeneous samples. This approach has unique applications in studying starch, insoluble proteins, and food microstructure, among others. One major application of ssNMR in foods is the analysis of starch‐based materials, which are abundant in cereals, tubers, and processed products. Native starch granules are semi‐crystalline, containing both ordered (crystalline) and disordered (amorphous) domains. Solid‐state ^13^C NMR can clearly distinguish these domains. For example, the C‐4 carbon of the glucose units in starch gives separate resonances for crystalline versus amorphous environments (typically, a sharp peak around 82–84 ppm for crystalline regions and a broader signal around 84–86 ppm for amorphous regions; Barison et al. 2022). By deconvoluting such signals, ssNMR allows quantification of starch crystallinity, which is directly related to qualities like digestibility and texture (more crystalline starches are often less digestible and contribute to the resistant starch fraction). Solid‐state NMR is also invaluable for monitoring starch gelatinization and retrogradation. During processes like cooking or bread staling, starch undergoes phase transitions: ssNMR has been used in situ to follow the conversion of crystalline amylopectin to amorphous form upon heating and the slow re‐crystallization (retrogradation) upon cooling/storage (Nowacka‐Perrin et al. 2022). These molecular‐level observations correlate with macroscopic changes (e.g., bread staling leading to firmness). In fact, ^2^H and ^13^C ssNMR studies of bread have shown that amylopectin recrystallization is a key driver of staling, connecting NMR data with texture measurements (Nowacka‐Perrin et al. 2022). Solid‐state NMR uniquely addresses the study of insoluble proteins and aggregates in food. Many food proteins (gluten in wheat dough, casein in cheese, soy protein fibrils, etc.) form insoluble networks or complexes. Such systems are not amenable to solution NMR due to a lack of mobility, but ssNMR can probe their structure. For instance, wheat gluten, which forms the viscoelastic network in dough, has been examined by ^13^C and ^1^H MAS NMR both in dry and hydrated states. These studies revealed details of protein secondary structure (proportions of alpha‐helix vs. beta‐sheet) and how hydration plasticizes the gluten network. High‐resolution ssNMR on glutenin proteins indicated that hydration increases the mobility of certain segments, which is important for dough rheology (Alberti et al. 2002). More generally, ssNMR can detect protein conformational changes in foods—for example, distinguishing native vs. denatured states in powdered milk proteins or aggregated states in amyloid‐like protein fibrils in some food processing scenarios. Because many proteins in food (e.g., in seeds or baked products) are part of insoluble matrices, ssNMR is one of the few techniques to get molecular information from them. As one review noted, “many proteins are insoluble and can be studied only in the solid state” (Krushelnitsky and Reichert 2005). Solid‐state NMR provides that window, giving atomistic insights into protein structure in situ, such as protein–protein and protein–water interactions in a food matrix. Another rich area for ssNMR is the study of food biopolymers and fibers. Cellulose, pectin, and other dietary fibers are insoluble polysaccharides that strongly influence nutritional properties (like fiber fermentability) and textural traits. ssNMR can characterize their composition and rigidity. For example, the relative intensities of different ^13^C signals in cellulose (from glycosidic carbons) can indicate the ratio of crystalline cellulose I versus amorphous domains, which affect how fibers break down. Food packaging polymers that migrate into foods or encapsulate ingredients can also be analyzed by ssNMR to ensure stability and lack of interaction with the food. Solid‐state NMR is additionally valuable for studying water dynamics in semi‐solids. Using ^1^H wide‐line NMR or ^2^H NMR on deuterated samples, one can measure how water is bound or constrained in a gel or solid matrix. This has been applied to gelatin gels, high‐solid confectionery gels, and freeze‐dried products to optimize rehydration properties. Fat crystallization in foods (like cocoa butter polymorphs in chocolate) has also been studied: ^13^C ssNMR can distinguish different crystalline forms of fats, which is crucial for chocolate quality (the difference between a shiny, snappy chocolate and a bloom‐covered one often comes down to fat crystal form; El Nokab et al. 2022). In summary, solid‐state NMR extends the reach of magnetic resonance into the solid and semi‐solid realm of foods, shedding light on structures and interactions that define texture, stability, and nutritional quality. By studying starch crystallinity, protein networks, and other insoluble components, ssNMR provides a structural perspective that complements solution NMR and MRI. As food scientists seek to design foods with specific textures or controlled release of nutrients, the role of ssNMR in characterizing the underlying matrix at the atomic level is increasingly important.

### 5.6 Magnetic Resonance (MR) Applications in Dairy and Cheese Products

Use of magnetic resonance (MR) devices, among them nuclear magnetic resonance (NMR), magnetic resonance imaging (MRI), and electron spin resonance (ESR), has served as a game changer in the analysis, providing a highly accurate, non‐ invasive, and non‐destructive way to ensure quality, safety, and the origin of the food, sweets, and spirits one buys. Utilization of NMR and MRI has turned out to be very useful in the adulteration testing of dehydrated by‐products, composition identification, and quality change monitoring during its treatment and storage. In dairy product analysis, the NMR technique is used to rapidly and accurately detect adulterants in milk. The idea behind counterfeit has been achieved through water, synthetic milk, or chemical treatments like urea and hydrogen peroxide. A method that has gotten good results in the estimation of these adulterants is the Time‐Domain Nuclear Magnetic Resonance (TD‐NMR). For instance, transverse relaxation time (T1) measurements performed through TD‐NMR can tell what the genuine milk is from the faked ones by pointing and waving to the molecular dynamic movements (Balthazar, Guimarães, Rocha, et al. 2021; Balthazar, Guimarães, Silva, et al. 2021). Such capabilities extend to powdered milk, where TD‐NMR assesses fat content, moisture levels, and other critical quality parameters, providing rapid, solvent‐free alternatives to traditional chemical assays. High‐resolution ^1^H NMR spectroscopy further allows the identification of minor contaminants like melamine at trace levels, ensuring safety in milk powder formulations. In cheese products, NMR aids in profiling water‐soluble metabolites during maturation (Sørensen et al. 2022). ^1^H NMR has been able to identify the compounds lactic acid, acetic acid, and hydrophobic amino acids which induce good flavor and texture. These metabolites also play a key role as markers for ripening stages, with definite compounds given luck for example, short‐ and long‐aged cheeses. Amino acids such as lysine and phenylalanine have been attributed to the intermediate and prolonged ripening periods, sequentially. Furthermore, NMR‐based metabolomics gives a deep dive into the microbial communities in the cheese matrix, aiding in the product structure and quality acquisition. MRI offers complementary capabilities for structural and textural analysis of cheese (Afshari et al. 2020). In addition, a study by (Segura et al. 2024) utilized Magnetic Resonance Imaging (MRI) to analyze structural and textural changes during the ripening process of four Spanish sheep cheese varieties. MRI parameters, including The transverse relaxation time (T_2_) and the longitudinal relaxation time (T_1_) times, apparent diffusion coefficient maps, and proton density, were used to assess variations in cheese matrix structure, manufacturing methods (industrial vs. traditional), ripening times (2–180 days), and geographical origins. A significant interaction between ripening time and cheese variety influenced physicochemical and textural properties. Logarithmic regression models demonstrated strong correlations between MRI parameters, water content, water activity, and texture. In another study, high‐resolution spectral treatment was used to authenticate and classify dairy products using ^1^H NMR spectra of cheese triacylglycerols. Quantifying 178 peaks helped construct multivariate models to forecast fatty acid composition and categorize cheese by species, provenance, and variation. Several biomarkers, such as anteisopentadecanoic, butyric, α‐linolenic, myristoleic, rumenic, and vaccenic acids, were identified. This study shows the first effective identification and quantification of minor fatty acids in cheese triacylglycerols using ^1^H NMR, demonstrating its potential for fast and accurate dairy verification (Haddad et al. 2022). On the other hand, in the study by (Cais‐Sokolińska et al. 2022) investigated the water behavior in buttermilk cheese with added polymerized whey proteins (PWP). Four cheese variants were analyzed: control (BMC), whey protein concentrate (BMC/WPC), single‐heated PWP (BMC/SPWP), and double‐heated PWP (BMC/DPWP). Differential scanning calorimetry (DSC) showed that BMC/WPC and BMC/DPWP had the highest freezable water content and lowest unfreezeable water content. NMR analysis indicated longer relaxation times in BMC/WPC, suggesting differences in water mobility. Single‐heated PWP tripled stickiness, while double‐heated PWP doubled shear work, improving textural properties. Color analysis (Δ*E*) suggested higher consumer acceptability for PWP‐enhanced cheeses, highlighting their potential for improved quality and texture in dairy products. Additionally, in the study about metabolism, the PDO Grana Padano cheese authenticity model was developed using NMR and multivariate analysis. A targeted study that isolated lactate, amino acids, and lipids revealed Grana Padano PDO's typical metabolic shape, which the untargeted method identified. The procedure's low variation and complete analysis of rare shredded and imitatation cheeses are its main benefits. This helps identify contaminated cheeses. The study found NMR to be a new and effective PDO cheese protection technology. MRI can track water distribution and microstructural changes to monitor ripening in real time. Proton relaxation dynamics from raw, heat‐treated, and pasteurized milk can help identify cheese. The NMR method on Pecorino and Fiore Sardo cheeses was successful, so it could be used for regulatory and authenticity checks (Maestrello et al. 2024). Moreover, the study by (Xu et al. 2022) examined the influence of fermentation time on the nutritional composition and spectroscopic properties of cheese over 25 and 50 days. Physical and chemical analysis showed that water, fat, protein, and soluble sugar content significantly decreased (*p* < 0.01) in 50‐day cheese, while hardness and chewiness increased (*p* < 0.01). Fourier Transform Infrared (FT‐IR) spectroscopy revealed differences in protein structure between the two fermentation stages. NMR analysis indicated early‐stage free water loss, with bound water dominating in later stages. Ultraviolet–visible (UV–Vis) spectroscopy identified distinct absorption peaks in ethyl acetate and petroleum ether extracts, linked to α, β‐unsaturated acids, esters, amides, and flavor‐associated aldehydes. These findings suggest that spectral properties can serve as characteristic indicators for cheese fermentation monitoring.

If we look at dairy products, NMR has played a significant role in guaranteeing that the products are genuine and of good quality by helping to detect impurities like fake milk, urea, or hydrogen peroxide. Furthermore, NMR checks fat content, the area from which the milk is taken, and water mobility in dairy matrices, thus making it a tool of quality control completely (Balthazar, Guimarães, Rocha, et al. 2021; Balthazar, Guimarães, Silva, et al. 2021). Milk is the backbone of our diet. Nevertheless, it is among the most adulterated food items in the market. The most usual fraudulent methods are using milk from several species, diluting with water, or adding exogenous substances like salts, sugars, and melamine to make it look or taste different. ^1^H TD‐NMR (proton time‐domain nuclear magnetic resonance) has been confirmed as a valuable and prompt way to identify and measure milk adulteration, such as the incorporation of whey, tap water, synthetic milk, synthetic urine, urea, and hydrogen peroxide. A study involving 78 milk samples revealed that relaxation times significantly differed between authentic and adulterated samples, correlating with the degree of adulteration (Nascimento, Santos, et al. 2017). Regression models based on multivariate and univariate approaches demonstrated reliable predictions with standard errors of 2.34% and 3.79%, respectively. Classification models like SIMCA and KNN also showed high accuracy in distinguishing control and adulterated samples, achieving sensitivity and specificity rates between 0.66 and 1.00. Low‐field NMR has also been used to differentiate fresh milk from aged milk stored at 30°C for 96 h. Measurements of relaxation times offered insights into the molecular dynamics of water. The study found that as fresh milk aged, total moisture content initially decreased, followed by an increase, driven by shifts in bound and free water dynamics during the process (Greer et al. 2018). The chemical composition of milk powder includes lactose, proteins, fats, minerals, and essential vitamins such as A, B, and D. Time‐Domain Nuclear Magnetic Resonance (TD‐NMR) and stoichiometry have been utilized to quantify the fat content in various milk powder samples without requiring solvents. A study using multivariate modeling of T2 decay determined fat content in solid milk powder to range between 0.75% and 26% (g/100 g), while moisture content was found to range from 1.9% to 3.9% (Nascimento, Barsanelli, et al. 2017). In another study, NMR was employed to conduct a comprehensive analysis of whole‐fat goat milk powder (GMP), identifying 44 metabolites categorized into fat, sugar, and aromatic regions. The principal signals included aliphatic (0.5–3.5 ppm) and aromatic (6.0–9.5 ppm) metabolites such as creatine, carnitine, lactose, galactose, lecithin, and sucrose. Weaker signals, corresponding to metabolites like lactic acid and equuric acid, were linked to bacterial contamination, highlighting their importance in assessing GMP quality and safety (Sanchez et al. 2020).^1^H NMR has also been applied to identify water‐soluble metabolites in cheese, such as Alpine Asiago, at various maturation stages. Lactic acids were identified as primary signals in the ^1^H NMR spectrum, alongside other strong signals like citric acid, ethanol, glycerol, hydrophobic amino acids, and 2,3‐butanediol, a marker for cheese ripeness, as it disappears during maturation. Furthermore, lysine content was associated with medium‐to‐short ripening periods, whereas phenylalanine was an indicator of longer ripening durations. These findings demonstrated the effectiveness of ^1^H NMR in distinguishing cheese samples by ripening stage (Segato et al. 2019). Additionally, the high‐resolution NMR methods like 400 MHz NMR and 700 MHz HR‐MAS NMR have played a crucial role in the detection of melamine, a toxic contaminant. These methods can also be used to measure the concentrations of melamine in infant formulas and to reveal the presence of contamination at the sub‐milligram per kilogram level. The research proves that NMR can find use as a primary method for the investigation of foodstuffs and also of confectionery, including candy. Thereupon, both ^1^H and ^13^C NMR are indispensable tools that made it real to trace step by step the authenticity of milk through the determination of the original species of the raw milk. For the same token, the ^13^C NMR fatty acid profile of milk was successfully used to differentiate between milk obtained from various animals like cows and buffaloes. The identification of the profiles of the triglycerides from the milk of buffaloes fed in various Italian regions was performed in one study; this enabled a 100% correct determination. Chemometric methods such as PCA and DA were applied to verify this hypothesis, and subsequently the results were really promising as they had classification accuracies of more than 94.5% for the test samples. Table 4 presents various Magnetic Resonance (MR) techniques and their applications in the analysis of dairy and cheese products. Finally, High‐resolution NMR can simultaneously quantify multiple milk/cheese metabolites (organic acids, amino acids, lipids, etc.), enabling rapid authentication and ripening monitoring in a single experiment. Importantly, NMR screening of dairy is inherently non‐destructive and requires minimal sample preparation. MRI adds complementary information by non‐invasively mapping cheese microstructure (moisture and fat distribution) and tracking textural changes over time. Because MR preserves the sample, the same batch can be assessed repeatedly, yielding dynamic structural and metabolic insights throughout maturation without extensive processing. These multi‐component, spatially resolved MR methods thus offer a uniquely comprehensive platform for dairy quality control, adulteration detection, and ripening studies (Ebrahimnejad et al. 2018; Vlasiou 2023).

**TABLE 4:** MR applications in dairy and cheese products.

MR type	Application	Food product	Objective	References
NMR	Detection of adulterants	Milk	Identify synthetic milk, water, urea, and hydrogen peroxide	Santos et al. (2016)
TD‐NMR	Fat and moisture content analysis	Milk Powder	Assess fat and moisture levels	Nascimento, Barsanelli, et al. (2017)
^1^H NMR	Metabolite profiling	Goat Milk Powder	Identify metabolites like lactose, creatine, and carnitine	Sanchez et al. (2020)
NMR	Ripening marker identification	Cheese	Analyze lactic acid, acetic acid, and hydrophobic amino acids	Segato et al. (2019)
ESR	Oxidative stability	Dairy Spreads	Detect lipid oxidation and assess antioxidant efficacy	Kristensen et al. (2002)
HR NMR	Detection of melamine	Infant Formula	Quantify toxic melamine contamination	Bergana et al. (2019)
NMR	Species differentiation	Milk	Differentiate cow, goat, and buffalo milk by triglyceride content	Eltemur et al. (2023)
^1^H NMR	Aging analysis	Cheese	Measure 2,3‐butanediol as a marker of cheese ripeness	Kandasamy et al. (2020)
Low‐field NMR	Fat and moisture content analysis	Milk	Measurement of Fat and Protein Contents	Sørensen et al. (2022)

### 5.7 AI and Magnetic Resonance (MR) Applications in Food Analysis

The combination of Artificial Intelligence (AI) with Magnetic Resonance (MR) technologies has opened up new insights into the food analysis field by data processing improvement, predictive analytics, and the efficiency of quality control systems. This cooperation has proved to be very successful when it comes to food authentication, safety assurance, and nutritional profiling. AI algorithms have been proven to be effective in NMR spectroscopy for food authentication. Machine‐learning methods (e.g., Support Vector Machines, neural networks) and chemometric techniques like Principal Component Analysis (PCA) are being used to analyze complex NMR spectral data. For example, PCA—a statistical tool for reducing data dimensionality—can uncover patterns in spectra, while methods such as SVM then use those patterns to classify authentic vs. adulterated products. A typical example of this is wine, where AI‐augmented NMR was able to reach 95% or even higher for genuineness of the wines and for adulteration detection in olive oil (Binetti et al. 2017). These methods are now scalable for high‐throughput industrial applications, streamlining the process of ensuring food authenticity. In the food analysis, a study by (Al‐Habsi et al. 2024) sought to predict the magnitude of moisture, fat, and fatty acid in 14 fish species (free‐floating and non‐mobile) using low‐frequency nuclear magnetic resonance (LF‐NMR) and artificial intelligence (AI). To get the relationship between LF‐NMR proton relaxation parameters and fish composition, they carried out chemical analysis in the typical way. Linear regression for the models allowed the prediction of moisture (*p* < 0.00001), total fat (*p* < 0.0001), and some fatty acids, while the target variable's low coefficients (*R* ^2^: 0.224–0.490) indicated that they can be non‐linear functions. On the other hand, artificial intelligence models provided for decision tree regression with *R* ^2^: 0.780–0.694, General Regression Neural Networks (GRNN) with *R* ^2^: 0.847–1.000 (training), and 0.506–0.924 (validation). Then, a study by Yue Sun et al. explored the potential of starch‐based nanocomposites (SNCs) in food science, emphasizing their role in enhancing stability, bioactivity, and functionality. The integration of artificial intelligence (AI) and nuclear magnetic resonance (NMR) was examined to optimize SNC design, predict material properties, and improve production efficiency. AI‐driven modeling refines SNC formulations, while NMR provides high‐resolution structural insights, monitors stability, and elucidates molecular interactions in food matrices. Case studies demonstrated NMR's role in understanding bioactive compound behavior within SNCs. The review also highlighted AI‐assisted analytics in evaluating bioactivity and sensory attributes, underscoring the synergy between AI, NMR, and SNCs in developing advanced, high‐performance food formulations tailored to health and consumer preferences (Sun et al. 2025).

In food safety, AI‐powered MR systems have enabled rapid and sensitive detection of contaminants and pathogens. NMR‐based biosensors, integrated with AI, have been developed to identify microbial contamination, such as *Salmonella* in milk and *Pseudomonas* in beverages, with detection thresholds as low as 100 CFU/mL. These systems leverage AI to analyze real‐time NMR signals, significantly reducing the time required for food safety (Banerjee et al. 2019).

Deep learning models in food analysis have also optimized MR applications in metabolomics by facilitating the identification of biomarkers for freshness, spoilage, and nutritional value. Deep learning models analyze low‐field NMR data to predict moisture loss and texture degradation during food processing and storage. Such predictive models are vital for reducing waste and enhancing product quality (Ghosh and Datta 2023). A study by Leniak et al. introduced a machine learning approach using NMR spectroscopy data to predict logD values. Among tested models, Support Vector Regression with Bucket Integration achieved the best performance (RMSE 0.66), outperforming tools like JChem (0.91) and CplogD (1.27). The method proved efficient, low‐cost, and competitive with traditional molecular descriptors, showing strong potential for drug discovery and chemical analysis (Leniak et al. 2024). In addition, another study by Wang et al. highlighted the impact of AlphaFold2 (AF2), a learning‐based machine learning method, in predicting 3D protein structures from amino acid sequences. It emphasized that integrating AF2 with Nuclear Magnetic Resonance (NMR) data enhances model accuracy, particularly for flexible proteins. Given the gap between rapidly growing sequence data and limited experimentally resolved structures, the study proposed that combining NMR and AI offers a promising approach for improving protein modeling and accelerating drug discovery (Wang, Wen, et al. 2024). Moreover, the study by Azizan et al. investigated MD2 pineapple ( *Ananas comosus* ) waste extracts using ^1^H NMR‐based metabolomics and machine learning. Extracts from peel, crown, and core were tested for antioxidant and α‐glucosidase inhibitory activities. Crown extracts with 100% ethanol showed the highest DPPH and nitric oxide scavenging (IC50 = 296.31 and 338.52 μg/mL), while peel extract showed the strongest α‐glucosidase inhibition (IC50 = 92.95 μg/mL). Supervised machine learning, specifically Partial Least Squares (PLS) regression, was used to analyze NMR data and identify key bioactive metabolites (Azizan et al. 2020).

Moreover, AI‐based spectral boosting methods give an impetus to the sensitivity and precision of MR machines, which have been hampered by their traditional drawbacks such as low signal‐to‐noise ratios and long acquisition times. By way of illustration, sophisticated denoising methods contribute to the clarity, which i.e., enables the same level of precision; however, a faster scan can be conducted without losing data integrity. In this situation, the capacity is probably necessary for low‐field NMR, which is a less expensive type and a good fit for the industrial sphere of applications (Wang et al. 2023). Furthermore, AI and MR are working together in the field of foodomics to provide comprehensive information on food and health connections. AI technologies in the pre‐MR stage are producing information about metabolite interactions and are essential in making functional foods that are designed especially for individual dietary requirements. Thus, these innovations changed the situation in food science through the development of sonic, bigger, and stronger equipment for quality assurance and safety compliance (Miyazawa et al. 2022).

Table 5 shows cases the integration of Artificial Intelligence (AI) with Magnetic Resonance (MR) technologies for advanced food analysis. Applications include food authentication, pathogen detection, metabolomics, nutritional profiling, and structural analysis.

**TABLE 5:** AI‐enhanced MR applications in food analysis.

MR type	Application	Food product	Objective	References
AI‐NMR	Food authenticity	Wine	Authenticate grape variety and origin	Hategan et al. (2024)
NMR	Pathogen detection	Milk	Detect Salmonella contamination	Jin, Li, et al. (2020)
AI‐enhanced NMR	Metabolomics	Vegetables	Analyze texture degradation during drying	Cacciatore et al. (2023)
TD‐NMR	Shelf‐life prediction	Fruits	Assess moisture loss and freshness	Mahata et al. (2023)
AI NMR	Nutritional profiling	Functional Foods	Identify bioactive compounds	Doherty et al. (2021)
AI‐NMR	Contaminant detection	Beverages	Detect metal ions and other contaminants	Jahangiri and Orekhov (2024)
ESR	Lipid oxidation monitoring	Processed Foods	Predict lipid stability and shelf‐life	Pantić et al. (2022)
AI‐enhanced MRI	Structural analysis	Cheese	Monitor structural changes during ripening	Goyal and Goyal (2012)
AI—NMR	Food Analysis	Grapevine Variety and Wine	Analysis was to classify these varieties and region	Nyitrainé Sárdy et al. (2022)
TD‐NMR	Quality Monitoring	Orange juice	Monitoring of soluble pectin content	Bizzani et al. (2020)

## 6 Conclusion

The innovation of Magnetic Resonance (MR), which primarily involves Nuclear Magnetic Resonance (NMR), magnetic resonance imaging (MRI), and electron spin resonance (ESR), has made an indelible impact on the food science field by introducing adroit, non‐destructive, and precise methods for quality and safety control and reliability assurance. This think piece is intended to encapsulate the game‐changing realizations in the use of MR technology, the results of which prove its capability to detect contaminants, map the changes in structure and composition, and ensure food safety compliance with a guarantee. In the food industry, MR technologies have been in a class of their own when it comes to the detection of these substances and for the identification of the root causes and mechanisms behind these. Different methodologies, such as TD‐NMR and ^1^H NMR, have been successful in not only metabolites but also the moisture content of the products. The outcome has been verifying product quality and developing quality control protocols. In the same way, MRI has given us the ability to see the deep hidden changes in food textures and the relationship between these and the presence and absence of feeling in the mouth. The use of ESR for the detection of the lipid oxidation process has been very beneficial in supplying data to extend the life of the product as well as preserve its freshness. In other words, AI technology is incorporated into MR devices, which becomes a breakthrough for the analysis of food, gaining the ability to quickly and accurately process the data. All of these have been achieved by the use of AI algorithms, which trigger an increase in the sensitivity and scaling of the MR system leading to the creation of advanced functions such as the detection of contaminants of choice, tracking of biomarkers, and others. This linkage brought forth the phase of foodomics, which is the prediction of molecular profiles on the basis of the nutritional profile and the personalized dietary solutions thereof. The MR systems are in a perfect position to help with those issues as consumer demand for transparency, safety, and sustainability keeps increasing, while food innovation is being challenged and advanced continually. Combining the updated spectroscope and AI‐run analytics, the stage is set for scientists and practitioners to unlock new possibilities that will see the food industry speak of an assurance of quality, safety, and authenticity in a multitude of applications across the entire sector. The continuous development of MR technologies, boosted by the integration of different fields, assures that these technologies are going to provide new perspectives on food science that meet the requirements of consumers and regulatory frameworks as well.

## Author Contributions

**Zina T. Alkanan:** writing – review and editing. **Ammar B. Altemimi:** investigation (equal), project administration (equal), writing – original draft (equal). **Farhang H. Awlqadr:** investigation (equal), writing – original draft (equal). **Seyed Mohammad Bagher Hashemi:** writing – original draft (equal), writing – review and editing (equal). **Anubhav Pratap‐Singh:** writing – original draft (equal). **Qausar Hamed Alkaisy:** writing – review and editing (equal).

## Ethics Statement

The authors have nothing to report.

## Consent

The authors have nothing to report.

## Conflicts of Interest

The authors declare no conflicts of interest.

## Data Availability

The data that support the findings of this study are available from the corresponding author upon reasonable request.
